# Comparative analysis of deep sequenced methanogenic communities: identification of microorganisms responsible for methane production

**DOI:** 10.1186/s12934-018-1043-3

**Published:** 2018-12-20

**Authors:** Adam Pyzik, Martyna Ciezkowska, Pawel S. Krawczyk, Adam Sobczak, Lukasz Drewniak, Andrzej Dziembowski, Leszek Lipinski

**Affiliations:** 1 Institute of Biochemistry and Biophysics, Polish Academy of Sciences, Pawinskiego 5a, 02-106 Warsaw, Poland; 2 Laboratory of Environmental Pollution Analysis, Faculty of Biology, University of Warsaw, Miecznikowa 1, 02-096 Warsaw, Poland; 3 Institute of Genetics and Biotechnology, Faculty of Biology, University of Warsaw, Pawinskiego 5a, 02-106 Warsaw, Poland

**Keywords:** Metagenome, Methanogens, Methanogenesis pathways, Environmental communities, Deep sequencing, Biogas

## Abstract

**Background:**

Although interactions between microorganisms involved in biogas production are largely uncharted, it is commonly accepted that methanogenic *Archaea* are essential for the process. Methanogens thrive in various environments, but the most extensively studied communities come from biogas plants. In this study, we employed a metagenomic analysis of deeply sequenced methanogenic communities, which allowed for comparison of taxonomic and functional diversity as well as identification of microorganisms directly involved in various stages of methanogenesis pathways.

**Results:**

A comprehensive metagenomic approach was used to compare seven environmental communities, originating from an agricultural biogas plant, cattle-associated samples, a lowland bog, sewage sludge from a wastewater treatment plant and sediments from an ancient gold mine. In addition to the native consortia, two laboratory communities cultivated on maize silage as the sole substrate were also analyzed. Results showed that all anaerobic communities harbored genes of all known methanogenesis pathways, but their abundance varied greatly between environments and that genes were encoded by different methanogens. Identification of microorganisms directly involved in different stages of methane production revealed that hydrogenotrophic methanogens, such as *Methanoculleus*, *Methanobacterium, Methanobrevibacter, Methanocorpusculum* or *Methanoregula*, predominated in most native communities, whereas acetoclastic *Methanosaeta* seemed to be the key methanogen in the wastewater treatment plant. Furthermore, in many environments, the methylotrophic pathway carried out by representatives of *Methanomassiliicoccales*, such as *Candidatus* Methanomethylophilus and *Candidatus* Methanoplasma, seemed to play an important role in methane production. In contrast, in stable laboratory reactors substrate versatile *Methanosarcina* predominated.

**Conclusions:**

The metagenomic approach presented in this study allowed for deep exploration and comparison of nine environments in which methane production occurs. Different abundance of methanogenesis-related functions was observed and the functions were analyzed in the phylogenetic context in order to identify microbes directly involved in methane production. In addition, a comparison of two metagenomic analytical tools, MG-RAST and MetAnnotate, revealed that combination of both allows for a precise characterization of methanogenic communities.

**Electronic supplementary material:**

The online version of this article (10.1186/s12934-018-1043-3) contains supplementary material, which is available to authorized users.

## Background

Biogas is one of the most promising solutions for energy production associated with degradation of various types of agricultural and industrial wastes, such as food waste, animal manure, crops and wastewater sludge. Under natural conditions, methane emission occurs in diverse anaerobic environments, such as animal digestive tracts, peatlands, anaerobic digesters, wetlands, rice field soils, marine sediments and hydrothermal habitats [1].

**Fig. 1:**
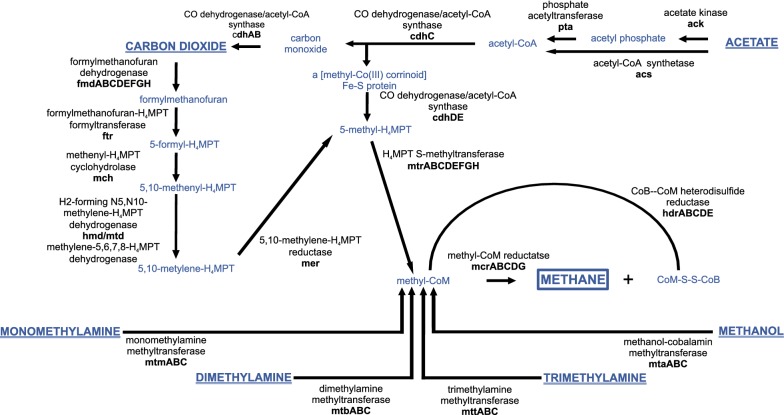
An overview of methanogenesis pathways from: carbon dioxide (hydrogenotrophic pathway); acetate (acetoclastic pathway); mono-, di-, tri- methylamine and methanol (methylotrophic pathway). Initial substrates for methane production and the final product were marked by bold capital letters. Additionally, initial substrates were underlined and the final product was marked with a frame

Based on the current knowledge, the conversion of organic matter into methane can be divided into four main steps: (i) hydrolysis; (ii) acidogenesis; (iii) acetogenesis and (iv) methanogenesis. The first three steps of biogas production can be carried out by a wide spectrum of microorganisms, but the final step, methanogenesis, is a limiting stage of the process, as it is performed exclusively by a group of *Archaea* called methanogens. Furthermore, methanogenesis can occur via three main pathways: (i) hydrogenotrophic (from carbon dioxide and hydrogen), (ii) acetoclastic (from acetate) and (iii) methylotrophic (from methylated compounds, such as methanol and methylamines) (Fig. 1). Regardless of the type of the methanogenesis pathway, its last common step involves the reduction of methyl-CoM into methane. The number of preceding stages is different for each methanogenesis pathway. In hydrogenotrophic and acetoclastic pathways carbon transfer occurs in six and three–four steps, respectively, while in the methylotrophic pathway only one class of enzymes (namely methyltransferases) is needed for the reduction of methylated compounds. Hydrogenotrophic and methylotrophic methanogenesis are more thermodynamically favorable than acetoclastic methanogenesis [2].

Many microorganisms responsible for the methanogenesis process have been identified and described so far. Most known biological producers of methane are represented within the *Euryarcheota* phylum: orders *Methanobacteriales*, *Methanocellales, Methanococcales*, *Methanomicrobiales*, *Methanopyrales*, *Methanosarcinales* and *Methanomassiliicoccales* [3, 4]. Most of methanogens carry out reduction of carbon dioxide connected with the consumption of hydrogen. However, within the orders of *Methanosarcinales*, *Methanobacteriales* and *Methanomassiliicoccales* there are microorganisms with the ability to convert methylated compounds into methane, while members of *Methanosarcinales* are capable of utilizing also acetate. Till now, only *Methanosarcina* of *Methanosarcinales* has been considered capable of carrying out all three methanogenesis pathways [4–7].

Development of metagenome sequencing allowed much better understanding of microorganisms’ role in anaerobic digestion and methane production by the analysis of both taxonomic and functional diversity [8–10]. In recent past, methanogenesis potential was often concluded from the abundance of a given methanogen only. However, genome-centric studies showed that conclusions drawn only from a taxonomic classification can lead to an underestimation of the true methanogenesis potential. For example, Rotaru and colleagues revealed that *Methanosaeta*, a genus assumed to be strictly acetoclastic, had a complete set of genes necessary for the reduction of carbon dioxide to methane via the hydrogenotrophic pathway [11].

Recent studies have suggested that we are only beginning to understand the diversity of methanogens and the methanogenesis process itself. The best studied methane-producing communities come from biogas reactors (e.g. [8–10]), but many waste materials used as a supplement in biogas systems could provide novel microorganisms adapted to the production of methane and the degradation of organic matter [12–15]. It seems right to assume that environmental communities are much more diverse but poorly characterized. This view has been reflected by the latest description of novel hydrogen-dependent methylotrophy in the *Methanomassiliicoccales* order [7] or by an identification of distant homologues of methanogenesis genes in *Bathyarchaeota*, which are still not considered methanogens [16]. Moreover there are recent reports demonstrating biogenic methane production by *Cyanobacteria* within the *Bacteria* domain which challenge the paradigm that methanogenesis is exclusive to *Archaea* [17].

The aim of this study was to characterize and compare the content of methanogenesis-related genes for very different anaerobic microbial communities which operates on diverse substrates and coming from methanogenic environments, such as agricultural biogas reactors (maize silage), a wastewater treatment plant (industrial and municipal wastes), cattle-associated habitats (grass and grains), a lowland bog (peat moss) and an ancient gold mine (decaying wooden rafters and other abandoned organic materials). With the use of widely available metagenomic tools, we performed an in-depth analysis of the methanogenesis process. Special focus was given to the identification of microorganisms directly involved in methane production in native communities by the phylogenetic classification of methanogenesis-related genes, especially for methyl-CoM reductase (*mcr*) that participates in the final step of methane release, as well as for formylmethanofuran dehydrogenase (*fmd*); CO dehydrogenase/acetyl-CoA synthase (*cdh*), methanol-specific methyltransferase (*mta*) and methylamine-specific methyltransferases (*mtm*, *mtb*, *mtt*) as they are responsible for triggering carbon dioxide (CO_2_), acetate (CH_3_COOH), methanol (CH_3_OH) and methylamines ((CH_3_)_x_NH)) utilization into methane, respectively [2, 4, 5, 8]. In addition to the deep-sequenced metagenome analysis, the methanogenesis potential was also assessed by a simple and commonly used method of semi-quantitative marker gene amplification [18].

This methodology was also applied to explore the taxonomic and functional community change after laboratory cultivation. The first laboratory community, originated from a fermenter tank of a biogas plant, was considered a model sample with an ability to produce biogas with methane content above 50% [19]. The second laboratory community came from raw sewage sludge from a wastewater treatment plant, as the sewage sludge is often used in biogas plants as a co-substrate source. In this type of samples, residual methanogenesis potential is usually low and methane content does not exceed 5% [20].

## Results

### General description of sequenced metagenomes and bioinformatic strategy

A metagenomic approach was used to characterize different anaerobic communities, and to assess their ability for methane production. Among the native communities, two originated from full-scale biogas reactors. Samples were collected from an agricultural biogas fermenter (ABF) and an agricultural biogas hydrolyzer (ABH) and were treated as a reference source of an efficient methanogenic community residing in a two-stage biogas plant. Aside from the extensively studied biogas reactor communities, we examined also more natural communities from environments where methane emission is detected. These other communities came from cattle manure (CM), cattle slurry (CS) and sewage sludge from a wastewater treatment plant (WTP), as biogas plants are often supplemented with these waste materials. The last two environmental samples originated from a lowland bog (LB) and an ancient gold mine (GM), as these habitats are a reservoir of diverse uncultivated microorganisms potentially beneficial to the methanogenesis process [21–23]. For comparative analyses of microbial community structural and functional change following a cultivation process, two laboratory consortia (ABF_TS and WTP_TS) were also implemented in the study. They were selected in batch reactors, and then stabilized in two-stage (TS) bioreactors.

**Fig. 2:**
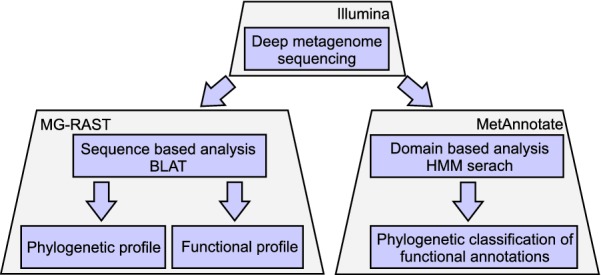
A schematic description of the metagenomic DNA analysis carried out in this study

Altogether, nine samples were analyzed in this study. Basic physico-chemical parameters were determined for all native and laboratory communities (see Additional file 1: Table S1) and metagenomic DNA was isolated and deep sequenced on the Illumina HiSeq 1500 platform. We obtained approximately 10–13 gigabases (79 million sequence counts on average) for each sample. The statistics of sequence counts and annotation analysis are presented in Additional file 1: Table S2. The reads obtained were analyzed using two different approaches implemented in the most commonly used pipelines (Fig. 2).

General analyses of community taxonomic and functional profiles were carried out through identification of similar sequences in reference databases with the use of the MG-RAST pipeline [24]. To obtain a more detailed insight into the methanogenesis process and the microbes involved, we used MetAnnotate [25], in which a HMM-based search is implemented and taxonomy is determined by the best hit or phylogenetic tree placement of obtained hits. In this study, we compared results from both approaches.

### Taxonomic profiling

**Fig. 3:**
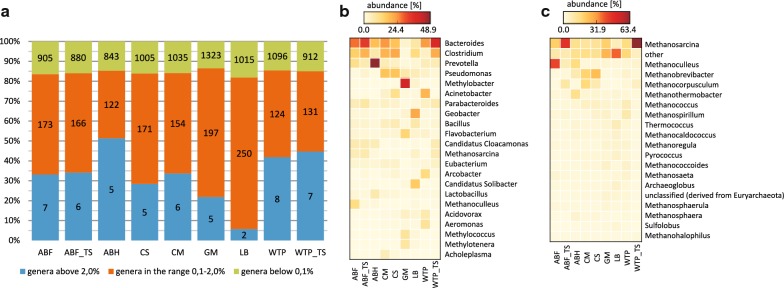
Microbial biodiversity of anaerobic consortia: **a** community structure of the analyzed consortia. Genera were binned according to their relative abundance in samples. The most abundant genera (> 2.0%), the least abundant genera (< 0.1%) and the intermediate ones (0.1–2.0%) are shown in blue, green, and orange, respectively. Numbers of genera in each abundance bin were included in the plot. **b** Relative abundance of the most abundant genera in the analyzed samples. Only genera with a sequence percentage greater than 2% in at least one metagenome are shown. **c** Relative abundance of the 20 most abundant archaeal genera in the analyzed samples considering *Archaea* sequences only. ABF—agricultural biogas plant fermenter; ABF_TS—laboratory reactor inoculated with agricultural biogas plant fermenter sample; ABH—agricultural biogas plant hydrolyzer; CS—cattle slurry; CM—cattle manure; GM—gold mine; LB—lowland bog; WTP—wastewater treatment plant; WTP_TS—laboratory reactor inoculated with wastewater treatment plant sample. All data were analyzed with MG-RAST, using RefSeq as the reference database

Taxonomic diversity was determined from metagenomic sequences annotated against the RefSeq database using the MG-RAST pipeline [24] (see Additional file 1: Table S3 and Tax MG-RAST, Additional file 2). At the domain level, all sequenced microbial communities were dominated by *Bacteria* (87.4–98.9%), followed by *Archaea* (0.4–11.5%) and *Eukaryota* (0.5–1.3%). Community structure analysis at the genus level showed 970 to 1525 different taxonomic groups in the samples. The most diverse and even was community originated from LB sample (Shannon–Wiener index—5.795, Pielou index—0.811) whereas the least versatile and uniform was ABH community (Shannon–Wiener index—4.055, Pielou index—0.590) (Additional file 1: Table S4). However in all samples there were only two to eight genera with the relative abundance above 2% (Fig. 3a). Microbes of *Bacteroides* and *Clostridium* genera were among the most common and the most abundant (Fig. 3b).

Among the investigated metagenomes, samples coming from agricultural biogas plant (ABF, ABH) were considered model consortia that harbor microorganisms essential for all steps of high-performance biogas production. The most specific feature of the agricultural biogas plant fermenter sample (ABF) was the high abundance of methanogenic archaeons, i.e. *Methanoculleus* (4.8%) and *Methanosarcina* (2.3%), whereas in other native metagenomes their abundance was significantly lower, close to 0.7% (ABH and LB samples) or even not exceeding 0.1% (CM, CS, GM and WTP samples (see Additional file 1: Table S3). Furthermore, in the agricultural biogas plant samples, we detected 2.6% (ABF) and 3.5% (ABH) of metagenomic sequences classified as *Candidatus* Cloacamonas, while in other native metagenomes sequence counts of this bacterium accounted for a maximum of 0.2%. Moreover, the ABH sample was highly enriched in polysaccharide-degrading *Prevotella* (28.5%), *Lactobacillus* (4.1%) and numerous genera belonging to *Veillonellaceae* family (2.2% cumulatively).

Interestingly, in the cattle-associated samples we observed that *Pseudomonas* (7.5%; 6.7%; CM and CS, respectively), *Acinetobacter* (1.5%; 4.2%) and *Bacillus* (2.4%; 2.2%) were more abundant than in bioreactor samples. In addition, CM was richer in members of *Acholeplasma* (2.4%). In the case of the community originated from a wastewater treatment plant (WTP), we observed a high number of sequences assigned to *Acinetobacter* (9.6%), *Arcobacter* (6.1%)*, Aeromonas* (4.6%) and *Acidovorax* (3.3%). The last two environmental samples analyzed, originating from a gold mine (GM) and a lowland bog (LB), were the most diverse and had few common genera with each other and with the remaining metagenomes (Fig. 3; Additional file 1: Fig. S1). The most unique feature of GM sample was a very high content of sequences assigned to methanotrophic *Methylobacter* (11.3%), *Methylococcus* (2.5%) and *Methylotenera* (2.3%). As a comparison, in other metagenomes these bacteria accounted for a maximum of 0.5%. In the case of LB community, the highest abundance was observed for *Geobacter* (3.3%) and *Candidatus* Solibacter (2.4%) (Fig. 3b). Importantly, LB community was enriched in numerous, low abundance environmental *Archaea*, including both methanogenic *Archaea* (3.9%) and other *Archaea* (4.7%) without a confirmed methane production ability (see Tax MG-RAST, Additional file 2).

Both laboratory consortia, originated from an agricultural biogas fermenter (ABF_TS) and sewage sludge from a wastewater treatment plant (WTP_TS), were dominated by *Bacteria* (93.0% in ABF_TS; 94.6% in WTP_TS), followed by *Archaea* (6.1% in ABF_TS, 4.9% in WTP_TS). Taxonomic annotations of the ABF_TS sample revealed that despite laboratory cultivation, the structure of microorganisms has been largely preserved (Fig. 3b; Additional file 1: Fig. S1). Nevertheless, we observed at least 1% change of the relative abundance of the following dominant genera: *Bacteroides* (from 10.9 to 14.1%), *Clostridium* (from 6.0 to 8.5%) and *Parabacteroides* (from 2.2 to 3.3%), *Pseudomonas* (from 0.3 to 1.3%), *Prevotella* (from 4.4 to 2.7%) and *Methanoculleus* (from 4.8 to 0.5%) (Fig. 3b; Additional file 1: Table S3). In the case of the consortium selected from sewage sludge of a wastewater treatment plant (WTP_TS), a substantial change of the community structure was observed compared to the native WTP sample (Fig. 3b; Additional file 1: Fig. S1). In general, abundance of *Proteobacteria* representatives diminished from 56.7 to 11.7%, while abundance of *Bacteroidetes* and *Firmicutes* increased from 26.8 and 10.0% in inocula to 34.6 and 33.8% in the laboratory consortium, respectively. Moreover, *Archaea* abundance increased from 0.5 to 4.9% of the total microbial structure. It seems that the most important change of laboratory consortium in comparison to its inoculum was the increase of fermentative *Bacteroides* (from 11.5 to 19.5%), *Clostridium* (from 2.1 to 10.7%), *Parabacteroides* (from 2.0 to 3.3%)*, Eubacterium* (from 0.8 to 2.1%), *Candidatus* Cloacamonas (from 0.1 to 3.1%), *Ruminococcus* (from 0.4 to 1.6%), *Syntrophomonas* (from 0.1 to 1.6%) and methanogenic *Methanosarcina* (from 0.1% to 3.1%) (see Additional file 1: Table S3). Simultaneously abundance of other genera such as *Acinetobacter*, *Arcobacter*, *Aeromonas*, *Acidovorax*, *Streptococcus*, *Flavobacterium*, *Thauera* and *Tolumonas* decreased from 9.6% in native community (WTP) to max. level of 0.6% in laboratory community (WTP_TS) (Fig. 3b; Additional file 1: Table S3).

### Functional profiling

To explore the metabolic potential of the studied communities, we performed a detailed analysis of metagenomic sequences annotated against SEED subsystems within the MG-RAST pipeline. We detected on average 5590 functional categories with at least 0.001% annotated reads (see Additional file 1: Table S2 and Fun MG-RAST, Additional file 2). Analysis of commonly used diversity and evenness indices showed that all metagenomes were quite similar in terms of diversity. The most functionally diverse and even was WTP community (Shannon–Wiener index—7.560, Pielou index 0.834) while the least one was ABF metagenome (Shannon–Wiener index—7.196, Pielou index 0.816) (see Additional file 1: Table S5). Furthermore, similarly to RefSeq Bray–Curtis distances calculation, some samples located near each other e.g. ABF and ABF_TS, while GM and LB samples were the most different from majority of the analyzed metagenomes (see Additional file 1: Fig. S2).

**Fig. 4:**
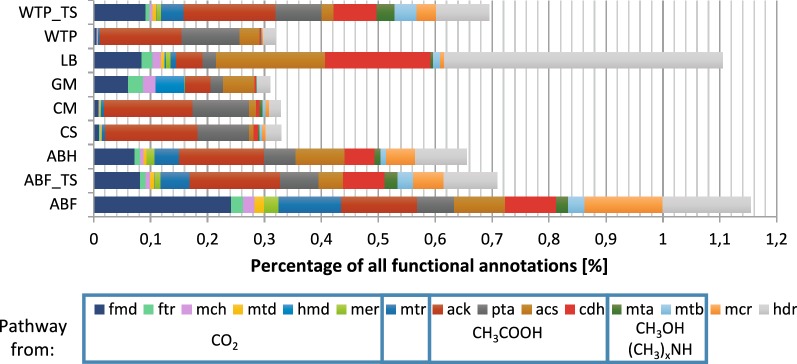
Relative abundance of methanogenesis-related genes shown as a percentage of total functional annotations of SEED subsystems analyzed by MG-RAST server. *fmd*—formylmethanofuran dehydrogenase (subunits ABCDEFGH); *ftr*—formylmethanofuran-H_4_MPT formyltransferase; *mch*—methenyl-H_4_MPT cyclohydrolase; *mtd*—methylene-5,6,7,8-H_4_MPT dehydrogenase; *hmd*—H_2_-forming *N*5,*N*10-methylene-H_4_MPT dehydrogenase; *mer*—5,10-methylene-H_4_MPT reductase; *mtr*—H_4_MPT-methyltransferase (subunits ABCDEFGHX); *ack*—acetate kinase; *pta*—phosphate acetyltransferase; *acs*—acetyl-CoA synthetase; *cdh*—CO dehydrogenase/acetyl-CoA synthase; *mta*—methanol-specific methyltransferase complex (subunits ABC); *mtb*—methylamine-specific methyltransferase complex (including subunits ABC for mono- di- and tri-methylamines utilization); *mcr*—methyl CoM reductase (subunits ABG); *hdr*—CoB-CoM heterodisulfide reductase (subunits ABCDE); H_4_MPT—tetrahydromethanopterin; CoA—coenzyme A; CoB—coenzyme B; CoM—coenzyme M. Genes involved in the given pathway, hydrogenotrophic (CO_2_), acetoclastic (CH_3_COOH) and methylotrophic (CH_3_OH and (CH3)_x_NH), were marked by frame. Genes common for all pathways were left without a frame. ABF—agricultural biogas plant fermenter; ABF_TS—laboratory reactor inoculated with agricultural biogas plant fermenter sample; ABH—agricultural biogas plant hydrolyzer; CS—cattle slurry; CM—cattle manure; GM—gold mine; LB—lowland bog; WTP—wastewater treatment plant; WTP_TS—laboratory reactor inoculated with wastewater treatment plant sample

We then focused on functional analysis relevant to methanogenesis pathways. Cumulative relative abundances of genes involved in various methanogenesis pathways were in the range of 0.31–1.15% (Fig. 4), with a caveat that genes from acetoclastic and methylotrophic pathways can contribute to processes other than methanogenesis.

Considering the percentage of methanogenesis-related annotations, ABF community followed by LB, ABF_TS and WTP_TS and ABH consortia had the highest potential for methane production via different pathways. Communities from an agricultural biogas plant and laboratory reactors (ABF, ABH, ABF_TS, WTP_TS) had similar sequence profiles of the selected genes. However, the functional profile of lowland bog (LB) metagenome differed significantly (Fig. 4). In the LB sample, we observed an overrepresentation of *acs*, *cdh* and *hdr* genes compared to other methanogenesis genes, including the key *mcr* genes. An even greater overrepresentation of genes involved in acetate utilization was observed for cattle-associated (CM, CS) and wastewater treatment plant (WTP) samples. However, in these samples most of the selected sequences were classified to *ack* and *pta* genes but few to *cdh* genes that directly mediate the transfer of carbon to methane pathway (Fig. 4). In the case of the native consortium from a gold mine (GM), we observed a relatively high number of sequences of *fmd, ftr, mch, hmd, ack, pta, acs* genes involved in the utilization of carbon dioxide and acetate, and very few sequences of *mcr* genes encoding the key methyl-CoM reductase enzyme.

The comparison of laboratory consortia and their native counterparts indicated that during laboratory cultivation the cumulative abundance of methanogenesis genes increased for WTP_TS and decreased for ABF_TS. In the case of WTP_TS, the increase concerned all genes except *ack*, *pta* and *acs*, while for ABF_TS the reduction concerned all studied genes.

### Methanogenesis genes-specific phylogenetic characterization

A sequence-based analysis of metagenomic sequences with the use of the MG-RAST pipeline gives an opportunity to explore metabolic potential of complex communities. Nevertheless, metagenomic data enables also a simultaneous identification of function and microorganisms responsible for specific processes. In the following part of our study, we present detailed results of phylogenetic placement of methanogenesis-related sequences. For this type of an analysis, the HMM search and the taxonomic classification approach implemented in MetAnnotate [25] were used. We focused on genes encoding methyl CoM reductase (*mcr*) responsible for the final release of methane, as well as on sequences of genes encoding enzymes which are considered crucial in the utilization of various substrates, such as: CO_2_—formylmethanofuran dehydrogenase (*fmd*); CH_3_COOH—CO dehydrogenase/acetyl-CoA synthase (*cdh*); CH_3_OH—methanol methyltransferase (*mta*); (CH_3_)_x_NH—methylamine methyltransferase (*mtm, mtb, mtt*). A summary of key microorganisms involved in specific steps of the methanogenesis superpathway for each analyzed sample is presented in Additional file 1: Fig. S3A–I.

**Fig. 5:**
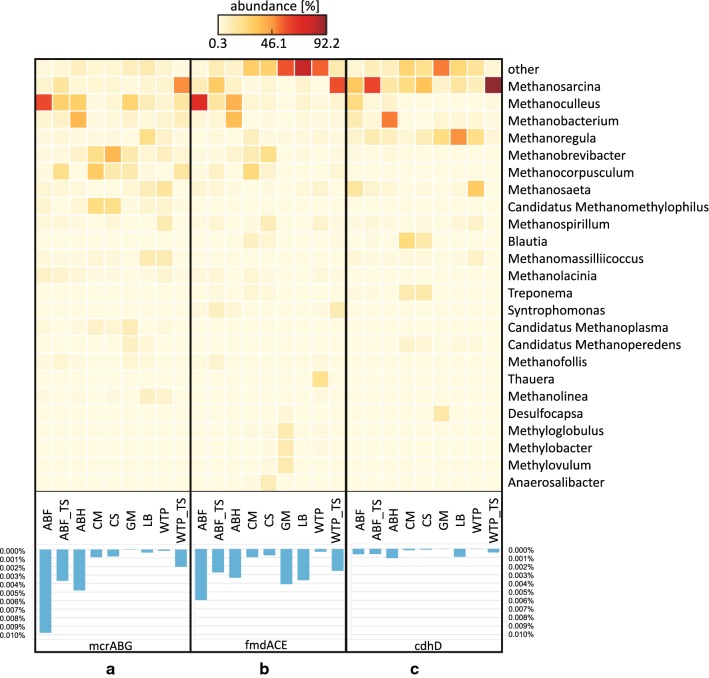
Taxonomic assignment at the genera level of the sequences of **a** methyl-CoM reductase (*mcrABG*); **b** formylmethanofuran dehydrogenase (*fmdACE*); **c** CO dehydrogenase/acetyl-CoA synthase (*cdhD*) obtained with MetAnnotate. The heatmap presents only genera with an abundance above 5% in at least two metagenomes or above 10% in one metagenome. The bar graph indicates the percentage of sequences detected in each metagenome. ABF—agricultural biogas plant fermenter; ABF_TS—laboratory reactor inoculated with agricultural biogas plant fermenter sample; ABH—agricultural biogas plant hydrolyzer; CS—cattle slurry; CM—cattle manure; GM—gold mine; LB—lowland bog; WTP—wastewater treatment plant; WTP_TS—laboratory reactor inoculated with wastewater treatment plant sample

The Metannotate-based analysis of native communities from an agricultural biogas plant (ABF and ABH) revealed that they were highly enriched in methyl-CoM reductase (*mcr)* genes. A high abundance of *mcr* genes was also observed in laboratory consortia (ABF_TS and WTP_TS) compared to the other environmental communities, such as CM, CS, GM, LB, WTP (Fig. 5a, bar graph panel). This is consistent with the data obtained using MG-RAST (Fig. 4). Metannotate-based analysis of *mcr* genes revealed substantial differences between analyzed samples in their phylogenetic placement. As was showed on Fig. 5a, most of the *mcr* reads of ABF sample were assigned to hydrogenotrophic *Methanoculleus* (59%), while in ABH they were dominated by *Methanobacterium* (39%) and *Methanoculleus* (30%). The largest number of *mcr* sequences in both of the laboratory communities (ABF_TS and WTP_TS) mapped to *Methanosarcina* (17%, 47%), *Methanoculleus* (29%, 16%) and *Methanocorpusculum* (22%, 16%), respectively. In the remaining metagenomes, most of the *mcr* sequences mapped also to hydrogenotrophic methanogens. In the cattle-related samples, *Methanobrevibacter* (23% in CM, 38% in CS) and *Methanocorpusculum* (31% in CM, 13% in CS) dominated, whereas *Methanoculleus* (20%) and *Methanoregula* (22%) dominated in GM and LB samples, respectively (Fig. 5a). In contrast, in WTP consortium, we observed a high number of *mcr* sequences mapped to acetoclastic *Methanosaeta* (17%). We also detected a substantial number of hits to representatives of the seventh order of methanogens, such as *Candidatus* Methanomethylophilus (24% in CM, 22% in CS) and *Methanomassiliicoccus* (12% in LB, 12% in WTP) (Fig. 5a).

Apart from the identification of microorganisms responsible for the last step of methanogenesis, we aimed for identifying those involved in carbon incorporation from different substrates, such as CO_2_, CH_3_COOH, CH_3_OH and (CH_3_)_x_NH. An analysis of formylmethanofuran dehydrogenase subunits ACE (*fmdACE*), indicators of hydrogenotrophic pathway, showed that among the native communities most hits were detected for ABF consortium, followed by GM, LB, ABH, ABF_TS, WTP_TS, CM, CS and WTP communities (Fig. 5b, bar graph panel). There was an up to 19-fold difference in the *fmdACE* abundance between the ABF and WTP samples. In the ABF sample, hydrogenotrophic *Methanoculleus* (66%) was the organism predominantly assigned to *fmd* genes. In the WTP sample, a majority of *fmd* sequences mapped to *Thauera* (21%) and to numerous taxa of *Archaea* and *Bacteria*, which did not exceed 6% (Fig. 5b). Following the laboratory cultivation of the aforementioned communities, *Methanosarcina* predominated in ABF_TS (28%) and WTP_TS (54%) *fmd* sequences. In comparison, for LB metagenome many of the *fmd* sequences mapped to various low-abundant *Archaea* and *Bacteria* with the highest counts for *Desulfobacter* (7%) and *Methylobacterium* (7%). Interestingly, in the case of GM metagenome, most *fmd* sequences were classified to methanotrophic bacteria, such as *Methylobacter*, *Methyloglobulus*, *Methylovulum* (12% each), but few were classified to methanogenic *Archaea* (Fig. 5b). In contrast, agricultural biogas hydrolyzer (ABH), cattle manure (CM) and cattle slurry (CS) were dominated mainly by hydrogenotrophic methanogens, such as *Methanoculleus* (35%) and *Methanobacterium* (32%) in ABH sample, *Methanocorpusculum* (23%) in CM sample and *Methanobrevibacter* (22%) in CS sample. Furthermore, both CM and CS had many hits (~ 10%) for *Methanospirillum*, *Methanoregula*, *Anaerosalibacter* and *Blautia* (Fig. 5b).

In order to identify microorganisms involved in the acetoclastic pathway, we analyzed phylogenetic assignments of the D subunit of CO dehydrogenase/acetyl-CoA synthase (*cdhD*), as it is directly involved in the transmission of a methyl group from acetate during acetoclastic methanogenesis [4, 5]. The abundance of *cdhD* was highest for ABH, followed by LB, ABF, ABF_TS, WTP_TS, CM, CS samples, while in GM and WTP metagenomes it was scarce (Fig. 5c, bar graph panel). Identification of phylogenetic matches of *cdhD* indicated that in most of the metagenomes acetate utilization was mediated by two to four dominant genera with percentage above 10%. In the case of laboratory communities, *cdhD* sequences were mostly assigned to one methanogen, namely *Methanosarcina,* which accounted for 65% (ABF_TS) and 94% (WTP_TS). In the case of the other two samples, approximately half of the annotations were classified to one methanogen, namely *Methanobacterium* (53%) in ABH and *Methanoregula* (49%) in LB (Fig. 5c). For other metagenomes, *cdhD* sequences were assigned to few dominant genera. These were *Methanosarcina* (32%), *Methanoculleus* (24%), *Methanosaeta* (17%), and *Methanobacterium* (12%) in the ABF metagenome; *Methanoregula* (24%) and *Desulfocapsa* (15%) in GM; *Methanosaeta* (31%) and *Methanoregula* (23%) in WTP and *Methanosarcina* (24%, 32%), *Blautia* (25%, 15%), *Treponema* (13%, 14%) in CM and CS samples, respectively (Fig. 5c). Furthermore, in the CM, CS, GM, LB, WTP samples, *cdhD* was often encoded by various *Archaea* and *Bacteria.*

**Fig. 6:**
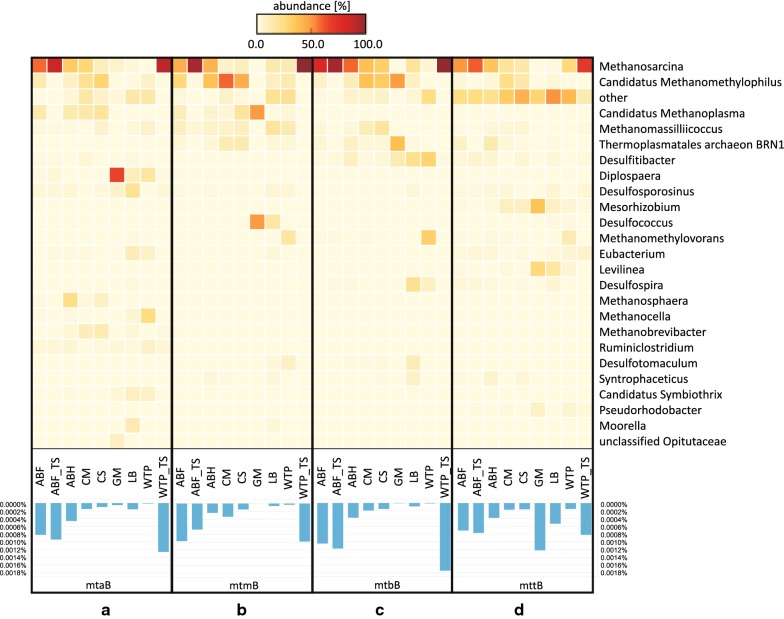
Taxonomic assignment at the genera level of the sequences of **a** methanol methyltransferase (*mtaB*); **b** monomethylamine methyltransferases (mtmB); **c** dimethylamine methyltransferases (*mtbB*); **d** trimethylamine methyltransferases (*mttB*) obtained with MetAnnotate. The heatmap presents only genera with an abundance above 5% in at least two metagenomes or above 10% in one metagenome. The bar graph indicates the percentage of *mtaB, mtmB, mtbB, mttB* sequences detected in each metagenome. ABF—agricultural biogas plant fermenter; ABF_TS—laboratory reactor inoculated with agricultural biogas plant fermenter sample; ABH—agricultural biogas plant hydrolyzer; CS—cattle slurry; CM—cattle manure; GM—gold mine; LB—lowland bog; WTP—wastewater treatment plant; WTP_TS—laboratory reactor inoculated with wastewater treatment plant sample

Utilization of methanol or methylamines is the third commonly recognized methanogenic pathway, which contains genes of methanol and mono-, di- and trimethylamine methyltransferases (*mta*, *mtm*, *mtb*, *mtt*, respectively). Comparison of the available domain profile sequences of *mtaB*, *mtmB*, *mtbB* and *mttB* showed that their abundances were almost constant between metagenomes, with the highest count for laboratory WTP_TS community and the lowest count for the native WTP consortium. The GM and LB metagenomes were an exception, as for them we detected enrichment of *mttB* compared to the other known methylotrophic domain profiles. Phylogenetic matches of functional annotations for genes of methylotrophic pathway showed that the majority of agricultural biogas plant, cattle-associated and laboratory sample sequences were assigned to *Archaea,* such as *Methanosarcina*, *Candidatus* Methanomethylophilus and *Candidatus* Methanoplasma (Fig. 6). The predominance of *Methanosarcina* (up to 99%) was particularly apparent for agricultural biogas plant and laboratory samples (ABF, ABH, ABF_TS, WTP_TS), while representatives of *Methanomassiliicoccales* were more abundant in CM and CS, for example, up to 60% of sequences of *Candidatus* Methanomethylophilus. In the case of GM, LB and WTP communities, we observed a higher contribution of bacterial genera, such as *Diplosphaera*, *Desulfococcus*, *Levilinea* and *Eubacterium* (even up to 66% of all annotations) (Fig. 6).

### Amplicon-based analysis of methanogenesis-related genes

In order to test if methanogenesis potential of deeply sequenced communities could be grasped by widely used and simple amplicon-based approach, we performed a semi-quantitative amplification of marker genes designed to monitor the methanogenesis potential of environmental samples [18]. PCR-amplified genes included alpha, beta and gamma subunits of methyl-CoM reductase (*mcrABG*), methanol-specific methyltransferase (*mtaB*) and methylamine-specific methyltransferase (*mtbA*).

**Fig. 7:**
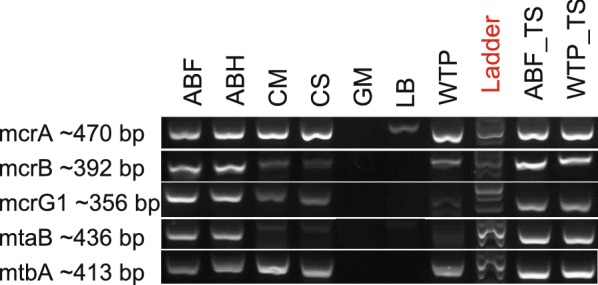
Comparison of methanogenesis gene profiles based on PCR products amplified on metagenomic DNA. *mcrA, mcrB, mcrG*—alpha, beta and gamma subunits of methyl-CoM reductase; *mtaB*—methanol-specific methyltransferase; *mtbA*—methylamine-specific methyltransferase; ABF—agricultural biogas plant fermenter; ABF_TS—laboratory reactor inoculated with agricultural biogas plant fermenter sample; ABH—agricultural biogas plant hydrolyzer; CS—cattle slurry; CM—cattle manure; GM—gold mine; LB—lowland bog; WTP—wastewater treatment plant; WTP_TS—laboratory reactor inoculated with wastewater treatment plant sample

Results showed that all five analyzed genes were successfully amplified from total DNA originating from ABF, ABH, CS, CM, WTP and laboratory communities ABF_TS and WTP_TS, which suggested their potential to carry out methane fermentation. However, lowland bog (LB) and gold mine (GM) communities seemed to have weak methanogenesis potential, as for the LB sample only *mcrA* product and a low amount (a faint band) of *mtaB* product were obtained and for the GM sample none of the PCR products were obtained (Fig. 7). In contrast, the control amplification of bacterial and archaeal 16S rDNA fragments was positive for all samples (Additional file 1: Fig. S4).

## Discussion

Metagenome analysis is a very useful approach for a comprehensive description of complex microbial communities. With various tools, a different level of insight into community performance may be gained. Here, we applied deep shotgun metagenomic sequencing coupled with two commonly used metagenomic analytical tools, MG-RAST and MetAnnotate, to describe and compare nine samples coming from the environments where methane production was detected. A more general description of taxonomic and functional structure, achievable with MG-RAST, was complemented with detailed analyses, including phylogenetic placement of methanogenesis-related genes, using MetAnnotate. Our approach enabled us also to compare applicability of both tools for metagenomic analysis of methanogenic environments.

MG-RAST-based diversity analysis, presented in this study, demonstrated that organic matter degraders, such as *Bacteroides, Clostridium*, *Parabacteroides*, *Prevotella*, *Candidatus* Cloacamonas, *Candidatus* Solibacter or *Geobacter*, predominated in native consortia. These microorganisms were previously detected in other methanogenic communities [26–35]. Both our results and literature data [36–39] suggest that high abundance of *Bacteroides* could increase overall carbohydrate hydrolytic performance. Despite an important role of organic matter degraders, the key players in biogas systems are methanogenic *Archaea,* which often constitute only a small fraction of a microbial community. Among the communities analyzed in this study, ABF was the most enriched in *Archaea*, followed by LB, ABF_TS, WTP_TS, ABH, CS, CM, WTP and GM (see Additional file 1: Table S3). *Archaea* abundance corresponded well with the proportions of functional annotations related to methanogenesis in different samples (Fig. 4). However, the GM metagenome had a relatively high abundance of genes of the hydrogenotrophic pathway despite a low abundance of *Archaea*.

Metagenomic analysis with the MG-RAST pipeline offered an insight into the *Archaea* community structure and the abundance of genes involved in methanogenesis. The identity of microbes involved in pathways cannot be, however, easily inferred without the use of additional tools. With such general approach (separate taxonomic and functional analyses), it is difficult to determine interactions between microorganisms involved in a given pathway, partially due to the dispersion of gene abundance in various low abundant microorganisms. In contrast, the second tool used in this study, MetAnnotate, allows a more detailed view and the identification of a specific function with the simultaneous assignment to a taxonomic group. In most cases in this study, results obtained with both tools were consistent. Microorganisms that were among the most abundant archaeons identified by MG-RAST dominated also among microorganisms with methanogenesis-related functions assigned by MetAnnotate. This result proved that some conclusions on the functional structure of a community can be drawn from a phylogenetic structure only. In the analysis of samples coming from a mesophilic agricultural biogas plant, the model sample of methanogenic community used in this study, both tools indicated that hydrogenotrophic *Methanoculleus* and substrate-versatile *Methanosarcina* were the main methanogens involved in methane production in ABF samples (see Additional file 1: Fig. S3A). In contrast, for ABH, the results obtained with different tools were less consistent. The diversity analysis by MG-RAST revealed that *Methanoculleus*, *Methanothermobacter* and *Methanosarcina* are the major *Archaea* in the analyzed sample (Fig. 3c), suggesting that they could be the major contributors to methane production. However, MetAnnotate showed that most of the methanogenesis-related sequences mapped to *Methanobacterium* and *Methanoculleus* (see Additional file 1: Fig. S3C). However, this difference in the annotations probably resulted from a database bias, as MG-RAST did not assign any sequence in any sample to *Methanobacterium* but rather to other genera within the *Methanobacteriaceae* family (Additional file 1: Table S1). As *Methanoculleus, Methanosarcina* and *Methanobacterium*, and not *Methanothermobacter*, are often identified in mesophilic biogas reactors [1, 9, 40–42], the results obtained with MG-RAST should be processed with caution.

MetAnnotate-based phylogenetic classification of functionally annotated sequences proved to be more informative also for communities from less studied environments. In the case of samples from cattle manure (CM) and cattle slurry (CS), both MG-RAST and MetAnnotate indicated that hydrogenotrophic *Methanobrevibacter* and *Methanocorpusculum* were the dominant methanogens. However, MetAnnotate showed also that microorganisms from the seventh order of methanogens, such as *Candidatus* Methanomethylophilus and *Candidatus* Methanoplasma, substantially contributed to the methylotrophic pathway and to the final release of methane, as they encode *mta, mtm, mtb, mtt, mcr* genes (see Additional file 1: Fig. S3D, E). Whereas the predominance of *Methanobrevibacter* in cattle digestive tract is supported by the results of other studies, *Methanocorpusculum* were rarely detected in rumen [15, 43–45]. Furthermore, the identification of *Candidatus* Methanomethylophilus and *Candidatus* Methanoplasma in cattle-derived samples in this study is in agreement with the recent detection of *Methanomassiliicoccales* in wetlands and gastrointestinal tracts of various animals [46]. The identification of *Methanomassiliicoccales* representatives in various environments suggests that the methylotrophic pathway may be more important than previously anticipated and can be carried out in a hydrogen-dependent manner [7].

In the case of very diverse environmental communities, such as samples from a gold mine (GM) or a lowland bog (LB), results derived from the MG-RAST analysis showed that the abundance of methanogenesis genes differs, but it is difficult to infer which microorganisms are involved in methane production, because many microbes were present in an even and low abundance. As a comparison, MetAnnotate offers a direct assignment of microbes to a specific function. Methanogenesis-related sequences of GM and LB communities mapped to multiple genera of both *Archaea* and *Bacteria*, suggesting that methane production in GM and LB samples could be negatively affected by substrate competition or the need for an interspecies intermediate transmission. It seems that hydrogenotrophic *Methanoregula* and *Methanoculleus* and representatives of the seventh order of methanogens were the major contributors to methane production in the lowland bog (see Additional file 1: Fig. S3G) and in the gold mine (see Additional file 1: Fig. S3F), respectively. However, the classification of many sequences to microorganisms that can use methane as a substrate, such as *Archaeoglobus* in LB sample and methanotrophic bacteria (e.g. *Methylomonas*) in GM sample, suggested that in these environments methane could be immediately utilized [47, 48]. Additionally, high abundance of hydrogenothropic-pathway genes may suggest that methylated compounds are rapidly utilized as a source of carbon, rather than used for methane production. This is in agreement with the previous study showing that methylothrophic bacteria can be responsible for carbon assimilation and cycling in a gold mine environment [49].

The last native community analyzed in this study was isolated from sewage sludge of a wastewater treatment plant. From the diversity analysis performed by the MG-RAST pipeline it could be concluded that *Methanosarcina* was one of the main methanogens in the WTP sample (Fig. 3c), however a domain profile search for methanogenesis-related genes with the use of MetAnnotate revealed that methane production was mediated mainly by *Methanosaeta* and *Methanomethylovorans* (see Additional file 1: Fig. S3H). This is in agreement with other studies [50, 51] and suggests the importance of acetoclastic and methylotrophic methanogenesis in industrial wastewater plants.

Aside from characterization of native communities, we compared the results for laboratory communities ABF_TS and WTP_TS. Both MG-RAST and MetAnnotate analyses suggested that after laboratory cultivation, a functional and taxonomic change occurred both for ABF_TS and WTP_TS. It seems that the selection of microbial community structure could be linked to the operating conditions of laboratory reactors, with the type of substrate as the presumably most important factor. Substrate impact on microbial diversity and dynamics were showed elsewhere [33]. In our study, a drastic reorganization of the microbial structure occurred for WTP_TS community (see Additional file 1: Figs. S1, S2), probably due to organic material change from mixed protein:carbohydrates:lipids in natural environment of wastewater treatment plant to solely carbohydrates in laboratory bioreactors. In contrast, the operating conditions were similar for native ABF and laboratory ABF_TS, however some community profile change was also observed. It seems that, in both ABF_TS and WTP_TS samples, methane production was mediated mainly by *Methanosarcina* (see Additional file 1: Fig. S3B, I). However, ABF_TS retained the higher biodiversity of methanogens. While the dominance of *Methanosarcina* in laboratory reactors was previously demonstrated (e.g. [41, 42, 52]), our methodology enabled us to follow the changes by a direct, methanogenesis-specific taxonomic profiling.

Metagenomic approach proved to be effective in characterization of complex microbial communities, yet we also checked whether it is possible to gain insight into methanogenesis potential by a simple PCR-based amplification. The results showed that PCR amplification correctly predicts the presence of methanogenesis genes and thus the ability to produce methane by different methanogenic communities. However, it should be kept in mind that a PCR reaction is highly dependent on primer sequences and that—due to incompleteness of reference databases—not all sequences of environmental microbes could be detected by this method. Nevertheless, PCR amplification seems to be a convenient and simple method for a rapid, preliminary assessment of methanogenesis potential of communities coming particularly from well-studied habitats e.g. industrial reactors. For less studied and more diverse environments we suggest a more complex analysis of deeply sequenced metagenomes, as it offers the opportunity to identify even the low abundant microbes that contribute to methane emission.

It is important to note that due to the ongoing development of public databases and thus incompleteness of environmental microbial sequences there could still be microorganisms involved in methanogenesis process that are omitted in a metagenomic analysis. However, based on our results, metagenomic approach is the least biased method that could provide information about complex microbial communities, which are often very difficult to cultivate. As showed by Campanaro and colleagues [39], genome-centric metagenomic approach could shed even more light on syntrophic interactions of microorganisms. For a more comprehensive overview of environmental processes such as organic matter degradation and methanogenesis, other meta-omic approaches, such as metatranscriptomic and metaproteomic ones, should be employed alongside metagenomic studies [53–56].

## Conclusions

The approach presented in this study allowed to explore in detail complex microbial communities coming from methane-producing environments. Communities predisposed to efficient methane production would be expected to contain a high abundance of genes of different steps of hydrogenotrophic, acetoclastic and methylotrophic pathways, which optimally are encoded by a few microbes. This view held true for engineered environments, such as industrial biogas reactors or laboratory cultures, but in most of the native environments—which have rarely been studied so far—we observed different levels of methanogenesis genes and their dispersion amongst various microorganisms. This was especially apparent for the lowland bog community and could suggest that the less described habitats are reservoirs of little-known microorganisms that contribute to methane cycle. Additionally, in such natural communities, it is more essential to remove just enough intermediate metabolites via methane pathways to keep the community functional than to cover the whole methanogenesis pathway. In contrast, engineered communities are specifically selected for high methane yield capacity.

Still a lot of work has to be done for the comprehensive characterization of methanogenic communities. A general analysis by the MG-RAST pipeline proved to be useful, however, for less described environments with many microorganisms present in low abundance, inferring microbial contributions with MG-RAST could be problematic. A deeper insight into microbial interactions could be obtained by searching for domain sequences with the MetAnnotate pipeline, which links specific metabolic functions to a specific microorganism. Moreover, this phylogenetic assignment of methanogenesis annotations seems to work much better for native communities. As one example, MetAnnotate-based analysis of the methylotrophic pathway suggested that it is carried out by the seventh order of methanogens and may play an important role in methane production in diverse environments.

## Methods

### Environmental sample collection and DNA isolation

Microbial consortium samples analyzed in this study were collected from environments specialized in anaerobic digestion and methane production, such as (I) fermenter (ABF) and (II) hydrolyzer (ABH) tank of an agricultural biogas plant in Miedzyrzec Podlaski, Poland; (III) cattle slurry (CS) and (IV) cattle manure (CM) from a farm in Mikanow, Poland; (V) bottom sediments of effluents from an ancient gold mine (GM) in Zloty Stok, Poland; (VI) peat from a lowland bog (LB) in Otwock, Poland; (VII) raw sewage sludge from a wastewater treatment plant (WTP) “Czajka” in Warsaw, Poland. Semi-liquid samples, such as sludge from the agricultural plant, the wastewater treatment plant and the cattle slurry were collected after removal of the material located in the vicinity of a drain valve by the release of at least 20 L. Likewise for more stable samples, such as cattle manure and peat, an upper surface was excluded and the samples were collected from the depth of approximately 30 cm (manure) and 90 cm (peat). In the case of sample from the gold mine, the material was collected as bottom sediments and surrounding liquids. Where possible (without undesirable aeration), samples were thoroughly mixed. Samples comprised of solids and liquids were maintained under native conditions for a maximum of 16 h prior to DNA extraction. When it was not possible to maintain native conditions, the samples were stored in dry ice.

Isolation of metagenomic DNA was performed according to Dziewit et al. [18]. Briefly, 1 g of a sample biomass (crude sample of CM and LB, or pellet material after centrifugation of ABF, ABH, CS, GM, WTP samples) was resuspended in 2 mL of a lysis buffer (100 mM Tris–HCl (pH 8.0); 100 mM EDTA (pH 8.0); 100 mM Na_2_HPO_4_ (pH 8.0); 1.5 M NaCl; 1% (w/v) CTAB). Then the metagenomic DNA was extracted by a five-step bead-beating protocol, combined with freezing and thawing. The isolation of metagenomic DNA was performed in triplicate per sample. The final DNA purification from proteins, humic substances and other compounds was carried out using CsCl density gradient ultracentrifugation. The concentration and quality of the purified DNA were estimated using a NanoDrop 2000c spectrophotometer (NanoDrop Technologies) and by agarose gel electrophoresis.

### Amplicon amplification

The isolated whole community DNA (combined triplicates) was used as a template for amplification of methanogenesis markers as described by Dziewit and co-workers [18]. The amplified genes included methyl-CoM reductase (subunits mcrA, mcrB, mcrG1) as well as subunits of methanol-specific methyltransferase (mtaB) and methylamine-specific methyltransferase (mtbA). Primers used were: MLf/MLr; LMCRB/RMCRB; LMCRG1/RMCRG1; LMTAB/RMTAB; LMTBA/RMTAB [18, 57]. Additionally, as a control of polymerase chain reaction (PCR) and purity of each metanogenomic DNA, bacterial and archaeal 16S rDNA fragments were amplified (primers: S-D-Arch-0349-a-S-17/S-D-Arch-0786-a-A-20 and S-D-Bact-0341-b-S-17/S-D-Bact-0785-a-A-21) [58]. All PCR reactions were performed in a TProfessional Thermocycler (Biometra) with Phusion High-Fidelity DNA Polymerase (Thermo Scientific).

### Laboratory reactors operation

The cultivation experiment was carried out in lab-scale bioreactors with a working volume of 800 mL, made of 1 L GL 45 glass bottles (Schott Duran, Germany) connected with Dreschel scrubbers and 1 L Tedlar gas bags (Sigma, Germany) as a biogas collector. Batch reactors were inoculated with 10 g_vs_ L^−1^ of different methanogenic consortia and supplemented with 9.6 g_vs_ L^−1^ of maize silage. The bioreactors were filled up with spring low-mineral water to the working volume of 800 mL and then pH was adjusted to 7.2 with sodium carbonate. Following our preliminary studies, anaerobic digestion was performed at 37 °C for 21 days for twelve passages. Passaging was carried out every 21 days, with 20% (160 mL) of working volume of the new bioreactors (passages from 2 to 12) coming from the previous passage as an inoculum. Furthermore, batch cultivation was divided into two steps based on substrate input: (I) 9.6 g_vs_ L^−1^ of maize silage (passages 1–7) and (II) 28.8 g_vs_ L^−1^ of maize silage (passages 8–12) as the grading of substrate concentrations is a frequently used method for the adaptation of microbial communities. In order to stabilize the best performing consortia, a semi-continuous cultivation in two-stage bioreactors was performed. For this purpose, biomass remains from passages 8–12 were subsampled and further cultivated in batch reactors in order to achieve a sufficient amount of consortia for inoculation of two-stage biogas reactors and to accelerate the start-up phase of the process. Two-stage bioreactor was constructed according to the Polish Patent no. PL197595 [59]. The reactor was equipped with hydraulic agitation and operated in a quasi-continuous mode. The metagenomic DNA representing laboratory consortia was isolated after 30 days of cultivation in two-stage bioreactors. The isolation procedure was identical as described for environmental samples.

In all experiments, the bioreactors were fed with maize silage provided by a farm located in Mikanow, Poland. A bulk amount of maize silage was transported from Mikanow to the laboratory at room temperature, portioned into plastic bags, and stored at 4 °C.

### Analytical methods

To characterize the physico-chemical profiles of the studied environments, the following parameters were determined: methane content, volatile fatty acids (VFAs) content, total solids (TS) content, volatile solids (VS) content, chemical oxygen demand (COD) and pH. The TS and VS analyses were performed according to the American Public Health Association Standard Methods [60]. The VFAs content and COD were determined using Nanocolor^®^ kits (Macherey–Nagel, Germany). Methane content was analyzed by GC–MS gas chromatography (Agilent, USA).

### Library preparation and sequencing

Metagenomic DNA isolated from environmental and laboratory communities (combined triplicates) was used for library preparation with an Illumina TruSeq DNA Sample Preparation Kit according to the manufacturer’s protocol. Purifications of DNA fragments were performed with Agencourt AMPure XP beads (Beckman Coulter). The libraries were analyzed by electrophoresis on 2% agarose gels (1× TAE buffer) with GelGreen staining, 2100 Bioanalyzer (Agilent) High-Sensitivity DNA Assay and KAPA Library Quantification Kit for Illumina. The libraries obtained were sequenced on the Illumina HiSeq 1500 platform (HiSeq Reagent Kit v2, 300 cycles) in a pair-end mode with a read length of 150 bp.

### Sequenced data analysis

Metagenomic raw sequences were uploaded to Metagenomic Rapid Annotations using Subsystems Technology (MG-RAST) server [24]. The metagenomes used in this work are available under the project accession mgp16315. Taxonomic profiles of consortia were created against the RefSeq database and the functional profiles were generated using the matches to the SEED Subsystems database with default parameters.

Phylogenetic classification of functional annotations relevant to the methanogenesis process was performed using MetAnnotate [25] based on HMM search and phylogenetic placement and best-hit approach with default parameters. The available HMM profiles of PFAM [61] and TIGRFAM [62] protein families were included in the analysis for the following enzymes: formylmethanofuran dehydrogenase (PF02663, TIGR03121, TIGR03122); formylmethanofuran-tetrahydromethanopterin formyltransferase (PF01913); methenyltetrahydromethanopterin cyclohydrolase (TIGR03120); H2-forming *N*5,*N*10-methylene-tetrahydromethanopterin dehydrogenase (PF03201); methylene-5,6,7,8-tetrahydromethanopterin dehydrogenase (PF01993); 5,10-methylenetetrahydromethanopterin reductase (TIGR03555); acetate kinase (TIGR00016); phosphate acetyltransferase (TIGR00651); acetyl-CoA synthetase (PF16177); CO dehydrogenase/acetyl-CoA synthase (PF03598, PF03599, TIGR00314, TIGR00315, TIGR00381); tetrahydromethanopterin *S*-methyltransferase (PF04208, PF05440, PF04211, PF04207, PF04206, PF09472, PF04210, PF02007, TIGR01111, TIGR04166, TIGR01148, TIGR01112, TIGR01113, TIGR02507, TIGR01114, TIGR00314, TIGR00315, TIGR00381), methanol methyltransferase (PF12176); methylamine methyltransferase (PF05369, PF06253, PF09505, TIGR02368, TIGR02369); methyl-CoM reductase (PF02249, PF02745, PF02241, PF02783, PF04609, PF02505, PF02240, TIGR03256, TIGR03257, TIGR03259, TIGR03260, TIGR03264); CoB-CoM heterodisulfide reductase (TIGR03288, TIGR03290).

Phylogenetic assignments of methanogenesis sequences are listed in Tax-Fun MetAnnotate, Additional file 2. For better readability, key methanogenesis annotations were combined (see Tax-Fun MetAnnotate Combined, Additional file 2) and presented as heatmaps created with the Statistical Analysis of Metabolic Profile (STAMP) software [63].

Taxonomic diversity (Shannon–Wiener index, H) was calculated using diversity function from vegan package in R [64]. Eveness (J, Pielou index) was calculated using formula J = H/log(S), where H is Shannon–Wiener index and S is the species richness for given sample. Bray–Curtis distances were calculated using vegdist function from vegan package and processed using metaMDS function to produce multidimensional scaling (MDS) plots.

## Additional files

**Additional file 1: Table S1.** Physico-chemical characteristics of the studied samples. TS—total solids; VS—volatile solids; COD—chemical oxygen demand; VFA—volatile fatty acids; CH_4_—methane; #—data not available on site but obtained in laboratory by measuring the effectiveness of methane production from a given substrate. Experiments were performed in 1 L bottles with 10% of the substrate and 90% of mineral water in 37 °C for 21 days; **Table** **S2.** MG-RAST statistics of the analyzed metagenomes. Post QC—post quality control; *—percentage of the identified protein features in the predicted protein features; #—percentage of the identified functional categories in the identified protein features; **Table** **S3.** Microbial community structure build on protein annotations against the RefSeq database [%]. Only genera with abundance greater than 1% in at least one metagenome were shown. For better readability, Class and Order names were excluded from the table. E—*Euryarcheota*, A—*Acidobacteria*, B—*Bacteroidetes*, C—*Chloroflexi*, F—*Firmicutes*, P—*Proteobacteria*, S—*Spirochaetes*, T—*Tenericutes*, TH—*Thermotogae*, UB—unclassified *Bacteria*; **Table** **S4.** Shannon–Wiener diversity index and Pielou eveness measurement at genus level based on RefSeq annotations data from MG-RAST; **Fig.** **S1.** Multidimensional scaling plot of Bray–Curtis dissimilarity at genus level of RefSeq annotations data from MG-RAST. Samples in pairs of ABF and ABF_TS as well as CM and CS overlaps; **Table** **S5.** Shannon–Wiener diversity index and Pielou eveness measurement at function level based on Subsystem annotations data from MG-RAST; **Fig.** **S2.** Multidimensional scaling plot of Bray–Curtis dissimilarity at function level of Subsystem annotations analyzed by MG-RAST; **Fig.** **S3.** Overviews of methanogenesis pathways highlighting the key microorganisms (identified based on MetAnnotate assignments of the genes marked in red) for: (A) agricultural biogas fermenter (ABF); (B) laboratory reactor inoculated with the agricultural biogas fermenter sample (ABF_TS); (C) agricultural biogas hydrolyzer (ABH); (D) cattle manure (CM); (E) cattle slurry (CS); (F) gold mine (GM); (G) lowland bog (LB); (H) wastewater treatment plant (WTP); (I) laboratory reactor inoculated with the wastewater treatment plant sample (WTP_TS). Only genera with hits above 15% were shown with a name. In brackets, the number of microorganisms with hits in the range of 5–15% was indicated. The x sign indicates that sequences for a given enzyme were not detected in metagenomic data by MetAnnotate. The initial substrates for methane production and the final product were marked by bold capital letters. Additionally, initial substrates were underlined and the final product was marked with a frame; **Fig.** **S4.** PCR reaction control on isolated metagenomic DNA with primers specific to bacterial and archaeal 16S rDNA variable region V3–V4.

**Additional file 2.** Tax MG-RAST—General taxonomic analysis of RefSeq assignments of metagenomic sequences analyzed by MG-RAST; Fun MG-RAST—General functional analysis of Subsystem function assignments of metagenomic sequences analyzed by MG-RAST; Tax-Fun MetAnnotate—Methanogenesis specific phylogenetic assignments of metagenomic sequences analyzed by MetAnnotate; Tax-Fun MetAnnotate—Combined Consolidated key methanogenesis specific phylogenetic assignments of metagenomic sequences analyzed by MetAnnotate.
