# Supplementary material for: Comparative analysis of deep sequenced methanogenic communities: identification of microorganisms responsible for methane production
Source: Microb Cell Fact. 2018 Dec 20;17:197. doi: 10.1186/s12934-018-1043-3 (PMC6302309; doi:10.1186/s12934-018-1043-3)
Supplement: Supplementary file 1 — Additional file 1: Table S1. Physico-chemical characteristics of the studied samples. TS—total solids; VS—volatile solids; COD—chemical oxygen demand; VFA—volatile fatty acids; CH4—methane; #—data not available on site but obtained in laboratory by measuring the effectiveness of methane production from a given substrate. Experiments were performed in 1 L bottles with 10% of the substrate and 90% of mineral water in 37 °C for 21 days; Table S2. MG-RAST statistics of the analyzed metagenomes. Post QC—post quality control; *—percentage of the identified protein features in the predicted protein features; #—percentage of the identified functional categories in the identified protein features; Table S3. Microbial community structure build on protein annotations against the RefSeq database [%]. Only genera with abundance greater than 1% in at least one metagenome were shown. For better readability, Class and Order names were excluded from the table. E—Euryarcheota, A—Acidobacteria, B—Bacteroidetes, C—Chloroflexi, F—Firmicutes, P—Proteobacteria, S—Spirochaetes, T—Tenericutes, TH—Thermotogae, UB—unclassified Bacteria; Table S4. Shannon–Wiener diversity index and Pielou eveness measurement at genus level based on RefSeq annotations data from MG-RAST; Fig. S1. Multidimensional scaling plot of Bray–Curtis dissimilarity at genus level of RefSeq annotations data from MG-RAST. Samples in pairs of ABF and ABF_TS as well as CM and CS overlaps; Table S5. Shannon–Wiener diversity index and Pielou eveness measurement at function level based on Subsystem annotations data from MG-RAST; Fig. S2. Multidimensional scaling plot of Bray–Curtis dissimilarity at function level of Subsystem annotations analyzed by MG-RAST; Fig. S3. Overviews of methanogenesis pathways highlighting the key microorganisms (identified based on MetAnnotate assignments of the genes marked in red) for: (A) agricultural biogas fermenter (ABF); (B) laboratory reactor inoculated with the agricultural biogas fermenter sa [file 12934_2018_1043_MOESM1_ESM.docx]

**Additional Material**

**TITLE: Comparative analysis of deep sequenced methanogenic communities – identification of microorganisms responsible for methane production**

**AUTHORS:** Adam Pyzik^1^, Martyna Ciezkowska ^2^, Pawel S. Krawczyk^1^, Adam Sobczak^1,3^, Lukasz Drewniak^2^, Andrzej Dziembowski^1,3^, Leszek Lipinski^1^,

**AFFILIATION:**

^1^ Institute of Biochemistry and Biophysics, Polish Academy of Sciences, Pawinskiego 5a, 02-106 Warsaw, Poland;

^2^ Laboratory of Environmental Pollution Analysis, Faculty of Biology, University of Warsaw, Miecznikowa 1, 02-096 Warsaw, Poland;

^3^ Institute of Genetics and Biotechnology, Faculty of Biology, University of Warsaw, Pawinskiego 5a, 02-106 Warsaw, Poland

***Correspondence: lechu@ibb.waw.pl**

**Additional Information**

**Abbreviations:**

ABF – agricultural biogas plant fermenter

ABF_TS – laboratory reactor inoculated with agricultural biogas plant fermenter sample

ABH – agricultural biogas plant hydrolyzer

CS – cattle slurry

CM – cattle manure

GM – gold mine

LB – lowland bog

WTP – wastewater treatment plant

WTP_TS – laboratory reactor inoculated with a wastewater treatment plant sample

fmd – formylmethanofuran dehydrogenase

ftr – formylmethanofuran-H_4_MPT formyltransferase

mch – methenyl-H_4_MPT cyclohydrolase

mtd – methylene-5,6,7,8-H_4_MPT dehydrogenase

hmd – H_2_-forming N5,N10-methylene-H_4_MPT dehydrogenase

mer – 5,10-methylene-H_4_MPT reductase

mtr – H_4_MPT-methyltransferase

ack – acetate kinase

pta – phosphate acetyltransferase

acs – acetyl-CoA synthetase

cdh – CO dehydrogenase/acetyl-CoA synthase

mta – methanol methyltransferase complex

mtm – monomethylamine methyltransferase

mtb – dimethylamine methyltransferase

mtt – trimethylamine methyltransferase

mcr – methyl-coenzyme M reductase

hdr – CoB-CoM heterodisulfide reductase

H_4_MPT – tetrahydromethanopterin

CoA – coenzyme A

CoB – coenzyme B

CoM – coenzyme M

**Table S1**

Physico-chemical characteristics of the studied samples. TS – total solids; VS – volatile solids; COD – chemical oxygen demand; VFA – volatile fatty acids; CH_4_ – methane; # – data not available on site but obtained in laboratory by measuring the effectiveness of methane production from a given substrate. Experiments were performed in 1-L bottles with 10% of the substrate and 90% of mineral water in 37°C for 21 days.

|  | ABF | ABF_TS | ABH | CM | CS | GM | LB | WTP | WTP_TS |
| --- | --- | --- | --- | --- | --- | --- | --- | --- | --- |
| pH | 7.3 | 7.5 | 4.9 | 7.9 | 7.4 | 7.6 | 6.8 | 6.0 | 7.8 |
| Temp [°C] | 34 | 37 | 27 | 18 | 18 | 10 | 18 | 37 | 37 |
| TS [%] | 4.0 | 1.9 | 4.6 | 15.0 | 2.2 | 1.7 | 16.3 | 4.0 | 1.7 |
| VS [%] | 70.9 | 79.6 | 70.2 | 82.6 | 45.6 | 98.0 | 89.0 | 64.8 | 63.3 |
| COD [g/L] | 42.5 | 3.7 | 36.9 | 132.6 | 18.4 | 6.9 | 112.2 | 74.3 | 7.0 |
| VFA[g/L] | 7.4 | 2.0 | 5.4 | 2.0 | 11.3 | 58.5 | 31.9 | 11.9 | 1.6 |
| CH_4_ content [%] | 55 | 68 | 26 | 58^#^ | 65^#^ | 10 | 8 | 60^#^ | 61 |

**Table S2**

MG-RAST statistics of the analyzed metagenomes.

|  | ABF | ABF_TS | ABH | CM | CS | GM | LB | WTP | WTP_TS |
| --- | --- | --- | --- | --- | --- | --- | --- | --- | --- |
| Uploaded Sequences Count | 90591325 | 74879241 | 40076766 | 64959194 | 65536214 | 85498750 | 89576562 | 118468896 | 85490885 |
| Sequences Count after QC | 64403798 | 58913987 | 34285064 | 55797390 | 50535827 | 65042561 | 76015382 | 95991551 | 72032368 |
| Mean GC % | 48 ± 9 % | 44 ± 11 % | 44 ± 9 % | 40 ± 9 % | 40 ± 9 % | 47 ± 12 % | 51 ± 11 % | 46 ± 12 % | 45 ± 10 % |
| Predicted Protein Features | 40556959 | 38149949 | 18170269 | 39488554 | 34643852 | 47534147 | 63524499 | 68475978 | 48008296 |
| Identified Protein Features (*) | 8959258 (22.1%) | 10253926 (26.9%) | 5611165 (30.9%) | 12888230 (32.6%) | 10812485 (31.2%) | 12049946 (25.4%) | 10025059 (15.8%) | 27567701 (40.3%) | 14442960 (30.1%) |
| Identified Functional Categories (#) | 6207923 (69.3%) | 6957544 (67.9%) | 3349016 (59.7%) | 9108006 (70.7%) | 7698097 (71.2%) | 8933354 (74.1%) | 7818794 (78.0%) | 19855804 (72.0%) | 9046164 (62.6%) |
| Function level categories count above 0.001% | 5094 | 5610 | 5351 | 5556 | 5731 | 5571 | 5694 | 6248 | 5498 |

Post QC – post quality control; * – percentage of the identified protein features in the predicted protein features;
# – percentage of the identified functional categories in the identified protein features.

**Table S3**

Microbial community structure build on protein annotations against the RefSeq database [%]. Only genera with abundance greater than 1% in at least one metagenome were shown. For better readability, Class and Order names were excluded from the table. E – *Euryarcheota*, A – *Acidobacteria*, B – *Bacteroidetes*, C – *Chloroflexi*, F – *Firmicutes*, P – *Proteobacteria*, S – *Spirochaetes,* T – *Tenericutes*, TH – *Thermotogae,* UB – unclassified *Bacteria.*

| **Domain** | **Phylum** | **Family** | **Genus** | **ABF** | **ABF_TS** | **ABH** | **CM** | **CS** | **GM** | **LB** | **WTP** | **WTP_TS** |
| --- | --- | --- | --- | --- | --- | --- | --- | --- | --- | --- | --- | --- |
| ***Archaea*** | **E** | *Methanomicrobiaceae* | *Methanoculleus* | 4.8 | 0.5 | 0.7 | 0.0 | 0.0 | 0.0 | 0.2 | 0.0 | 0.2 |
|  |  | *Methanosarcinaceae* | *Methanosarcina* | 2.3 | 2.8 | 0.6 | 0.1 | 0.1 | 0.1 | 0.6 | 0.1 | 3.1 |
|  | other *Archaea* | | other | 4,4 | 2.8 | 3.0 | 1.0 | 0.9 | 0.4 | 8.1 | 0.4 | 1.6 |
| ***Bacteria*** | **A** | *Solibacteraceae* | *Candidatus Solibacter* | 0.3 | 0.2 | 0.1 | 0.1 | 0.1 | 0.3 | 2.4 | 0.1 | 0.1 |
|  |  | unclassified  *Acidobacteria* | *Candidatus Koribacter* | 0.1 | 0.1 | 0.1 | 0.0 | 0.0 | 0.2 | 1.4 | 0.0 | 0.1 |
|  | **B** | *Bacteroidaceae* | *Bacteroides* | 10.9 | 14.1 | 10.8 | 6.7 | 10.4 | 0.9 | 1.2 | 11.5 | 19.5 |
|  |  | *Porphyromonadaceae* | *Paludibacter* | 1.4 | 1.1 | 0.8 | 0.7 | 1.1 | 0.2 | 0.3 | 1.5 | 1.0 |
|  |  | *Porphyromonadaceae* | *Parabacteroides* | 2.2 | 3.3 | 1.6 | 1.3 | 1.9 | 0.2 | 0.3 | 2.0 | 3.3 |
|  |  | *Porphyromonadaceae* | *Porphyromonas* | 1.5 | 1.3 | 0.9 | 0.7 | 1.0 | 0.1 | 0.2 | 0.5 | 1.2 |
|  |  | *Prevotellaceae* | *Prevotella* | 4.4 | 2.7 | 28.5 | 1.7 | 2.5 | 0.2 | 0.3 | 2.7 | 2.9 |
|  |  | *Flavobacteriaceae* | *Flavobacterium* | 1.0 | 0.8 | 0.6 | 1.0 | 0.9 | 3.8 | 0.5 | 1.6 | 0.6 |
|  |  | *Sphingobacteriaceae* | *Pedobacter* | 1.1 | 0.7 | 0.6 | 0.5 | 0.6 | 1.1 | 0.4 | 0.3 | 0.6 |
|  |  | unclassified *Sphingobacteriales* | *Chitinophaga* | 0.6 | 0.4 | 0.3 | 0.2 | 0.2 | 1.0 | 0.5 | 0.2 | 0.3 |
|  |  | other *Bacteroidetes* | other | 8.7 | 6.8 | 4.8 | 5.7 | 6.0 | 8.4 | 3.5 | 6.3 | 5.1 |
|  | **C** | unclassified *Dehalococcoidete* | *Dehalococcoides* | 0.4 | 0.2 | 0.2 | 0.1 | 0.1 | 0.0 | 1.7 | 0.0 | 0.1 |
|  | **F** | *Bacillaceae* | *Bacillus* | 1.3 | 1.3 | 1.0 | 2.4 | 2.2 | 0.4 | 1.1 | 0.5 | 1.4 |
|  |  | *Lactobacillaceae* | *Lactobacillus* | 0.7 | 0.4 | 4.1 | 0.7 | 0.9 | 0.1 | 0.2 | 0.2 | 0.4 |
|  |  | *Streptococcaceae* | *Streptococcus* | 0.5 | 0.4 | 0.5 | 0.8 | 1.0 | 0.1 | 0.2 | 1.7 | 0.4 |
|  |  | *Clostridiaceae* | *Alkaliphilus* | 0.6 | 0.7 | 0.4 | 1.5 | 0.7 | 0.1 | 0.3 | 0.2 | 0.7 |
|  |  | *Clostridiaceae* | *Clostridium* | 6.0 | 8.5 | 4.4 | 8.5 | 8.7 | 0.4 | 1.9 | 2.1 | 10.7 |
|  |  | *Syntrophomonadaceae* | *Syntrophomonas* | 0.7 | 1.2 | 0.4 | 0.2 | 0.1 | 0.0 | 0.2 | 0.1 | 1.6 |
|  |  | *Ruminococcaceae* | *Ruminococcus* | 1.0 | 1.6 | 0.7 | 1.2 | 1.4 | 0.0 | 0.1 | 0.4 | 1.6 |
|  |  | *Eubacteriaceae* | *Eubacterium* | 1.1 | 1.2 | 1.4 | 1.5 | 1.6 | 0.0 | 0.2 | 0.8 | 2.1 |
|  |  | other *Firmicutes* | other | 12.4 | 13.5 | 11.8 | 13.1 | 13.5 | 1.4 | 8.0 | 4.2 | 15.0 |
|  | **P** | *Bradyrhizobiaceae* | *Bradyrhizobium* | 0.1 | 0.1 | 0.0 | 0.1 | 0.1 | 0.5 | 1.1 | 0.1 | 0.1 |
|  |  | *Bradyrhizobiacee* | *Rhodopseudomonas* | 0.1 | 0.1 | 0.1 | 0.1 | 0.1 | 0.5 | 1.0 | 0.1 | 0.1 |
|  |  | *Burkholderiaceae* | *Burkholderia* | 0.2 | 0.3 | 0.1 | 0.5 | 0.4 | 1.6 | 1.2 | 0.8 | 0.2 |
|  |  | *Comamonadaceae* | *Acidovorax* | 0.1 | 0.1 | 0.0 | 0.3 | 0.2 | 1.2 | 0.4 | 3.3 | 0.1 |
|  |  | *Comamonadaceae* | *Albidiferax* | 0.0 | 0.1 | 0.0 | 0.1 | 0.1 | 1.1 | 0.4 | 0.5 | 0.0 |
|  |  | *Comamonadaceae* | *Polaromonas* | 0.0 | 0.1 | 0.0 | 0.2 | 0.1 | 1.3 | 0.5 | 0.7 | 0.1 |
|  |  | *Methylophilaceae* | *Methylobacillus* | 0.0 | 0.0 | 0.0 | 0.1 | 0.1 | 1.2 | 0.2 | 0.1 | 0.0 |
|  |  | *Methylophilaceae* | *Methylotenera* | 0.0 | 0.0 | 0.0 | 0.1 | 0.1 | 2.3 | 0.2 | 0.1 | 0.0 |
|  |  | *Rhodocyclaceae* | *Dechloromonas* | 0.0 | 0.1 | 0.0 | 0.2 | 0.1 | 0.7 | 0.3 | 1.0 | 0.1 |
|  |  | *Rhodocyclaceae* | *Thauera* | 0.0 | 0.1 | 0.0 | 0.1 | 0.1 | 0.3 | 0.1 | 1.2 | 0.0 |
|  |  | *Desulfovibrionaceae* | *Desulfovibrio* | 0.7 | 0.6 | 0.3 | 0.3 | 0.3 | 0.3 | 1.0 | 0.6 | 1.3 |
|  |  | *Geobacteraceae* | *Geobacter* | 0.7 | 0.7 | 0.4 | 0.4 | 0.3 | 0.8 | 3.3 | 0.4 | 0.4 |
|  |  | *Myxococcaceae* | *Anaeromyxobacter* | 0.2 | 0.2 | 0.1 | 0.1 | 0.1 | 0.4 | 1.2 | 0.1 | 0.1 |
|  |  | *Campylobacteraceae* | *Arcobacter* | 0.0 | 0.6 | 0.0 | 1.1 | 0.6 | 0.1 | 0.1 | 6.1 | 0.5 |
|  |  | *Aeromonadaceae* | *Aeromonas* | 0.1 | 0.1 | 0.0 | 0.2 | 0.2 | 0.2 | 0.1 | 4.6 | 0.0 |
|  |  | *Aeromonadaceae* | *Tolumonas* | 0.0 | 0.0 | 0.0 | 0.1 | 0.1 | 0.1 | 0.0 | 1.0 | 0.0 |
|  |  | *Alteromonadaceae* | *Marinobacter* | 0.1 | 0.1 | 0.0 | 1.8 | 1.4 | 0.4 | 0.1 | 0.2 | 0.1 |
|  |  | *Shewanellaceae* | *Shewanella* | 0.3 | 0.3 | 0.2 | 1.3 | 1.1 | 1.0 | 0.4 | 0.9 | 0.2 |
|  |  | *Chromatiaceae* | *Nitrosococcus* | 0.1 | 0.1 | 0.1 | 0.3 | 0.3 | 1.2 | 0.4 | 0.1 | 0.1 |
|  |  | *Methylococcaceae* | *Methylobacter* | 0.1 | 0.1 | 0.0 | 0.1 | 0.1 | 11.3 | 0.5 | 0.1 | 0.2 |
|  |  | *Methylococcaceae* | *Methylococcus* | 0.1 | 0.1 | 0.0 | 0.1 | 0.1 | 2.5 | 0.3 | 0.1 | 0.1 |
|  |  | *Halomonadaceae* | *Chromohalobacter* | 0.0 | 0.0 | 0.0 | 1.5 | 1.1 | 0.1 | 0.1 | 0.0 | 0.0 |
|  |  | *Halomonadaceae* | *Halomonas* | 0.0 | 0.0 | 0.0 | 1.1 | 0.8 | 0.1 | 0.0 | 0.0 | 0.0 |
|  |  | *Moraxellaceae* | *Acinetobacter* | 0.1 | 0.2 | 0.1 | 4.2 | 1.5 | 0.4 | 0.2 | 9.6 | 0.1 |
|  |  | *Pseudomonadaceae* | *Pseudomonas* | 0.3 | 1.3 | 0.2 | 6.7 | 7.5 | 2.0 | 0.9 | 1.8 | 0.9 |
|  |  | *Xanthomonadaceae* | *Xanthomonas* | 0.1 | 0.1 | 0.1 | 0.3 | 0.2 | 1.1 | 0.3 | 0.5 | 0.1 |
|  |  | other *Proteobacteria* | other | 8.1 | 9.1 | 4.9 | 20.2 | 16.6 | 36.7 | 27.1 | 22.7 | 6.9 |
|  | **S** | *Spirochaetaceae* | *Treponema* | 0.8 | 1.1 | 0.3 | 0.4 | 0.4 | 0.0 | 0.2 | 0.1 | 0.6 |
|  | **T** | *Acholeplasmataceae* | *Acholeplasma* | 0.1 | 0.1 | 0.1 | 0.5 | 2.4 | 0.0 | 0.0 | 0.0 | 0.1 |
|  | **TH** | unclassified *Thermotogales* | unclassified *Thermotogales)* | 1.2 | 0.8 | 0.7 | 0.0 | 0.0 | 0.0 | 0.1 | 0.0 | 0.5 |
|  | **UB** | unclassified *Bacteria* | *Candidatus Cloacamonas* | 2.6 | 2.7 | 3.5 | 0.2 | 0.0 | 0.0 | 0.1 | 0.1 | 3.1 |
|  | other Bacteria | | | 14.4 | 13.2 | 9.4 | 6.8 | 6.8 | 9.6 | 23.3 | 5.1 | 9.8 |
| *Eukaryota* | | | | 1,0 | 0.9 | 0.5 | 0.7 | 0.6 | 1.4 | 1.4 | 0.5 | 0.6 |
| *Viruses* | | | | 0,1 | 0.0 | 0.1 | 0.2 | 0.2 | 0.0 | 0.0 | 0.1 | 0.0 |

**Table S4**

Shannon-Wiener diversity index and Pielou evenness measurement at genus level based on RefSeq annotations data from MG-RAST.

|  | ABF | ABF_TS | ABH | CM | CS | GM | LB | WTP | WTP_TS |
| --- | --- | --- | --- | --- | --- | --- | --- | --- | --- |
| Shannon-  Wiener index | 5.046 | 4.890 | 4.055 | 4.863 | 5.037 | 5.216 | 5.795 | 4.618 | 4.461 |
| Pielou index | 0.722 | 0.703 | 0.590 | 0.686 | 0.712 | 0.712 | 0.811 | 0.649 | 0.641 |

**
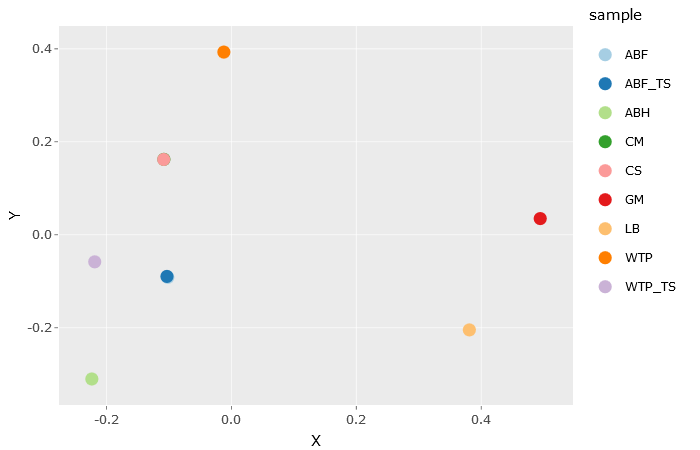
**

**Fig. S1**

Multidimensional scaling plot of Bray-Curtis dissimilarity at genus level of RefSeq annotations data from MG-RAST. Samples in pairs of ABF and ABF_TS as well as CM and CS overlaps.

**Table S5**

Shannon-Wiener diversity index and Pielou evenness measurement at function level based on Subsystem annotations data from MG-RAST.

|  | ABF | ABF_TS | ABH | CM | CS | GM | LB | WTP | WTP_TS |
| --- | --- | --- | --- | --- | --- | --- | --- | --- | --- |
| Shannon-  Wiener index | 7.196 | 7.306 | 7.259 | 7.381 | 7.434 | 7.400 | 7.327 | 7.560 | 7.310 |
| Pielou index | 0.816 | 0.818 | 0.828 | 0.823 | 0.828 | 0.832 | 0.820 | 0.834 | 0.818 |

**
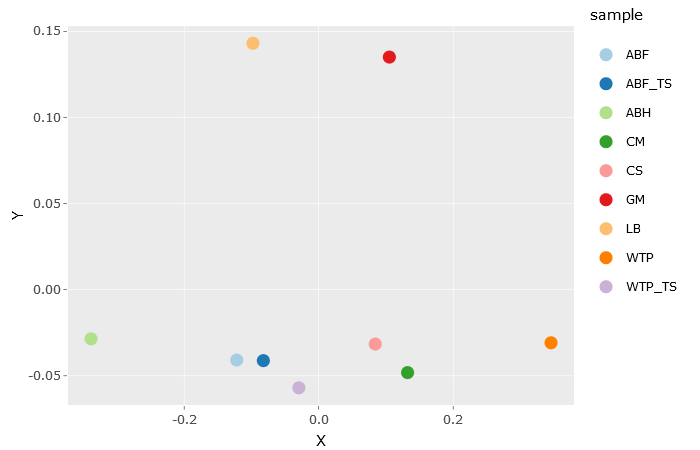
**

**Fig. S2**

Multidimensional scaling plot of Bray-Curtis dissimilarity at function level of Subsystem annotations analyzed by MG-RAST.

**
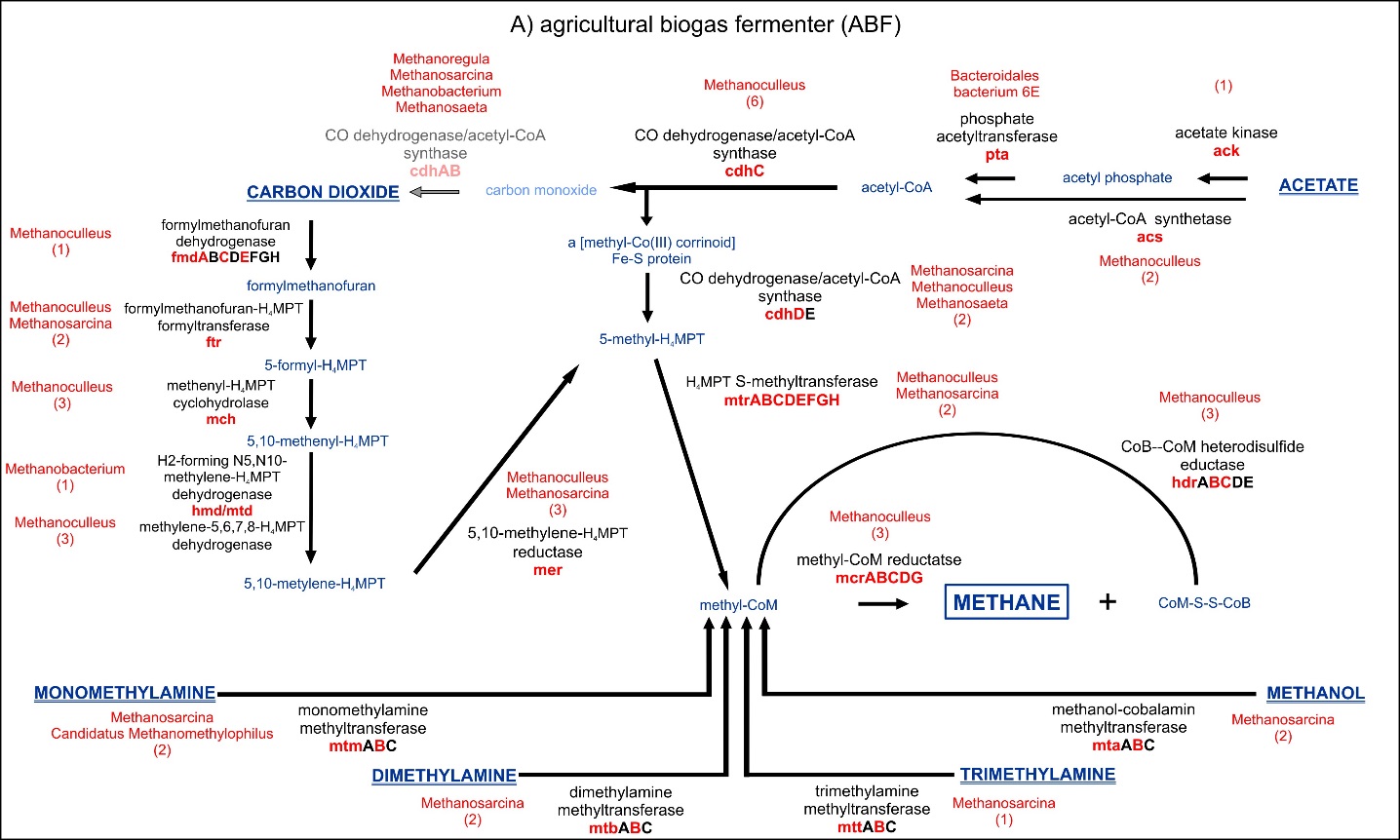

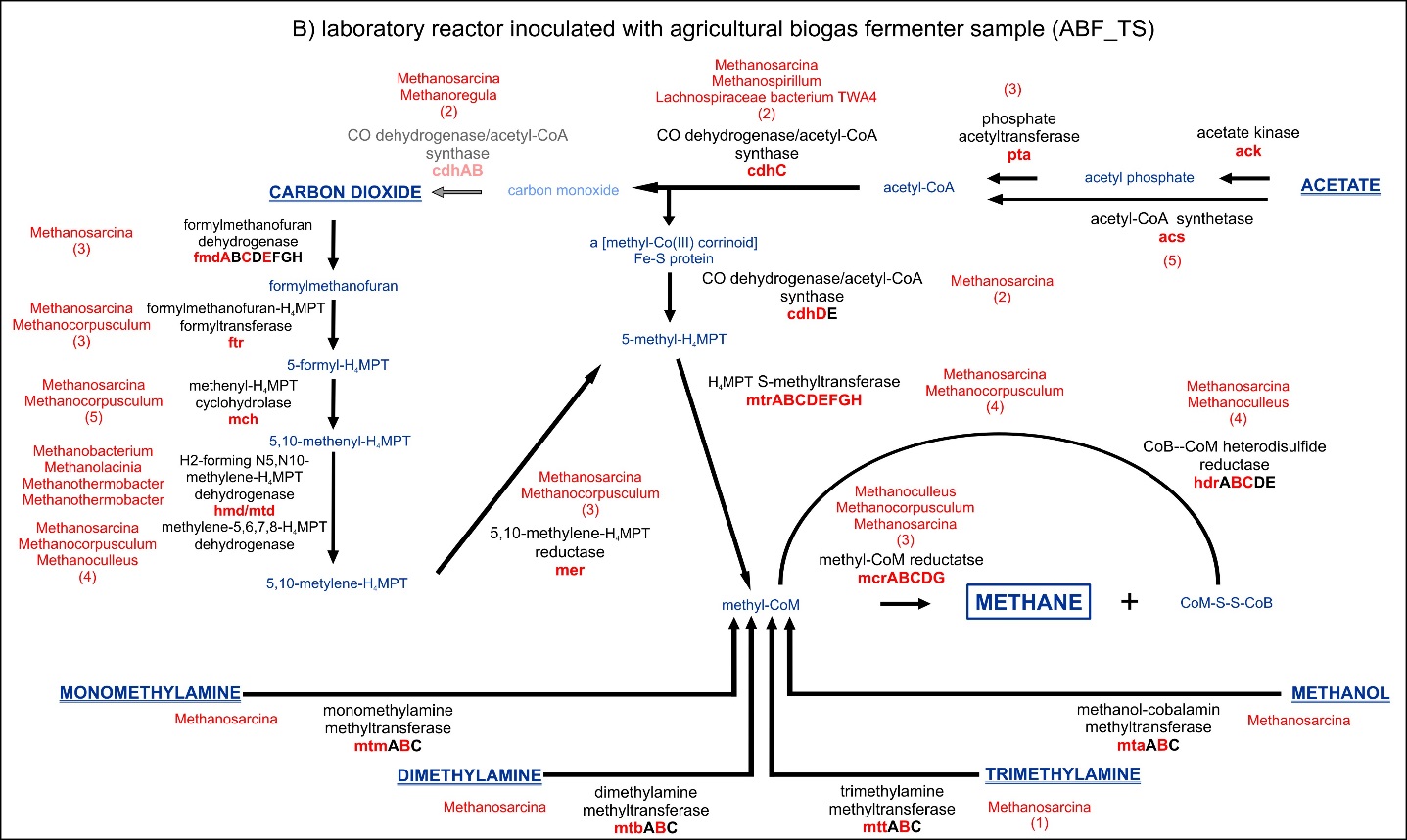
**

**
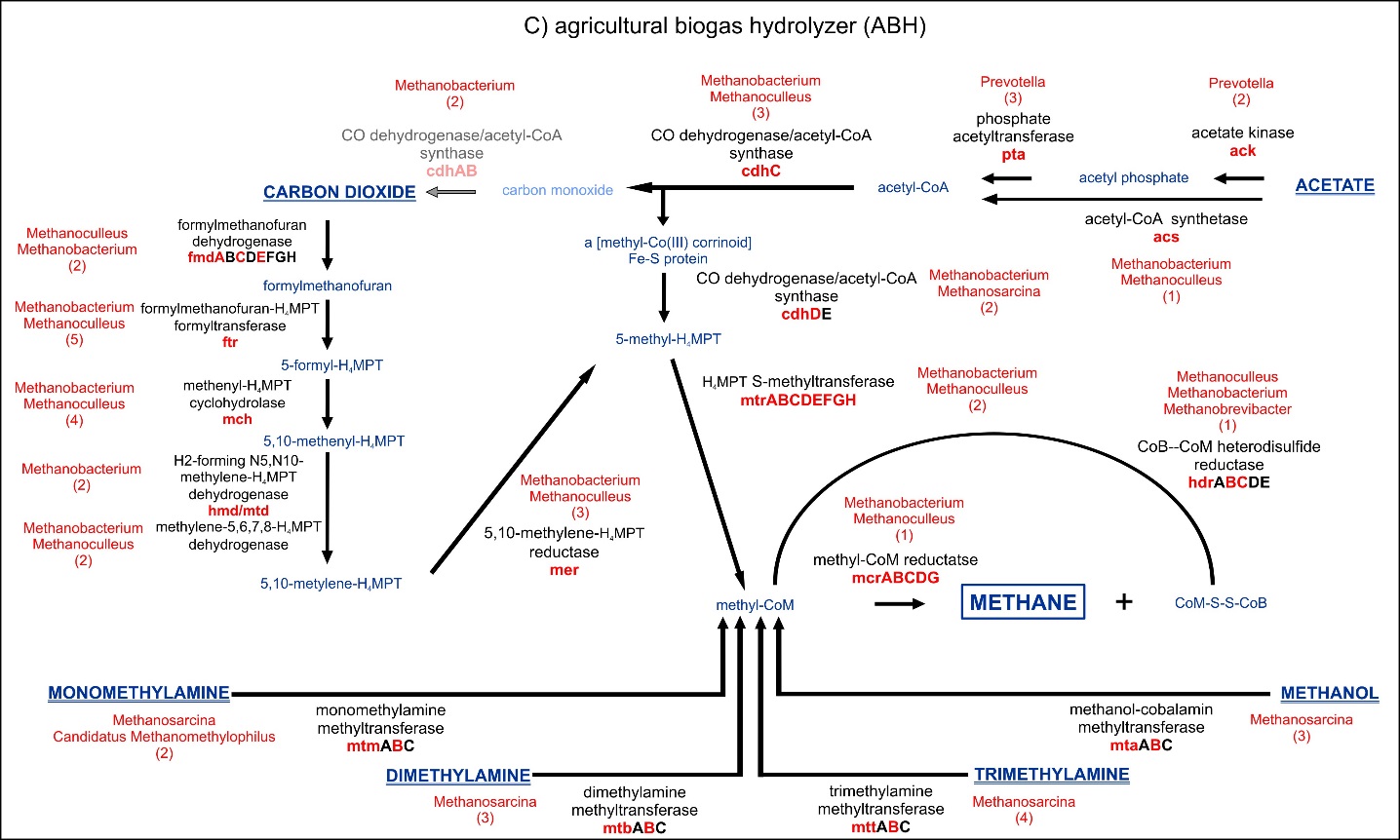
**

**
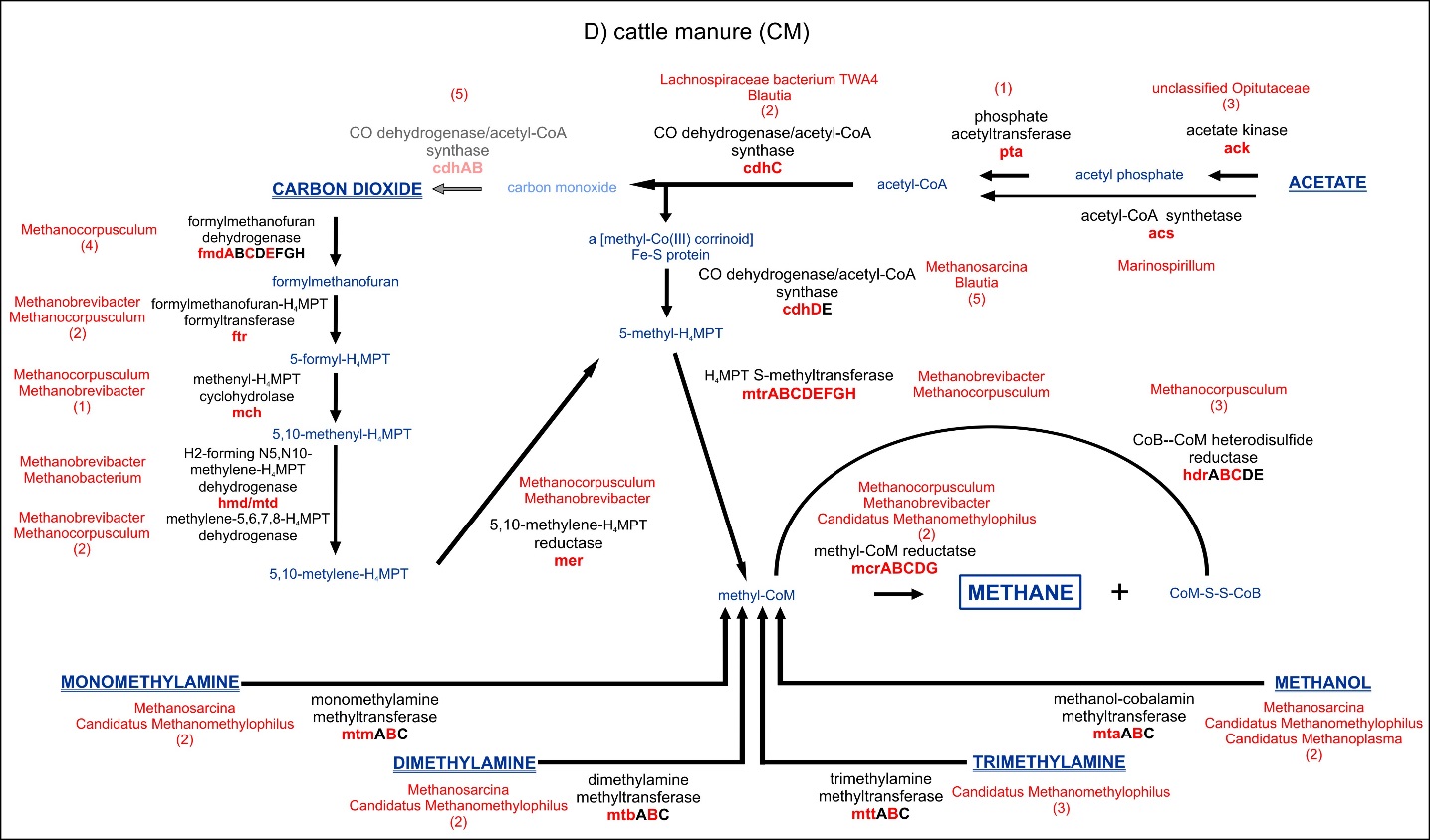
**

**
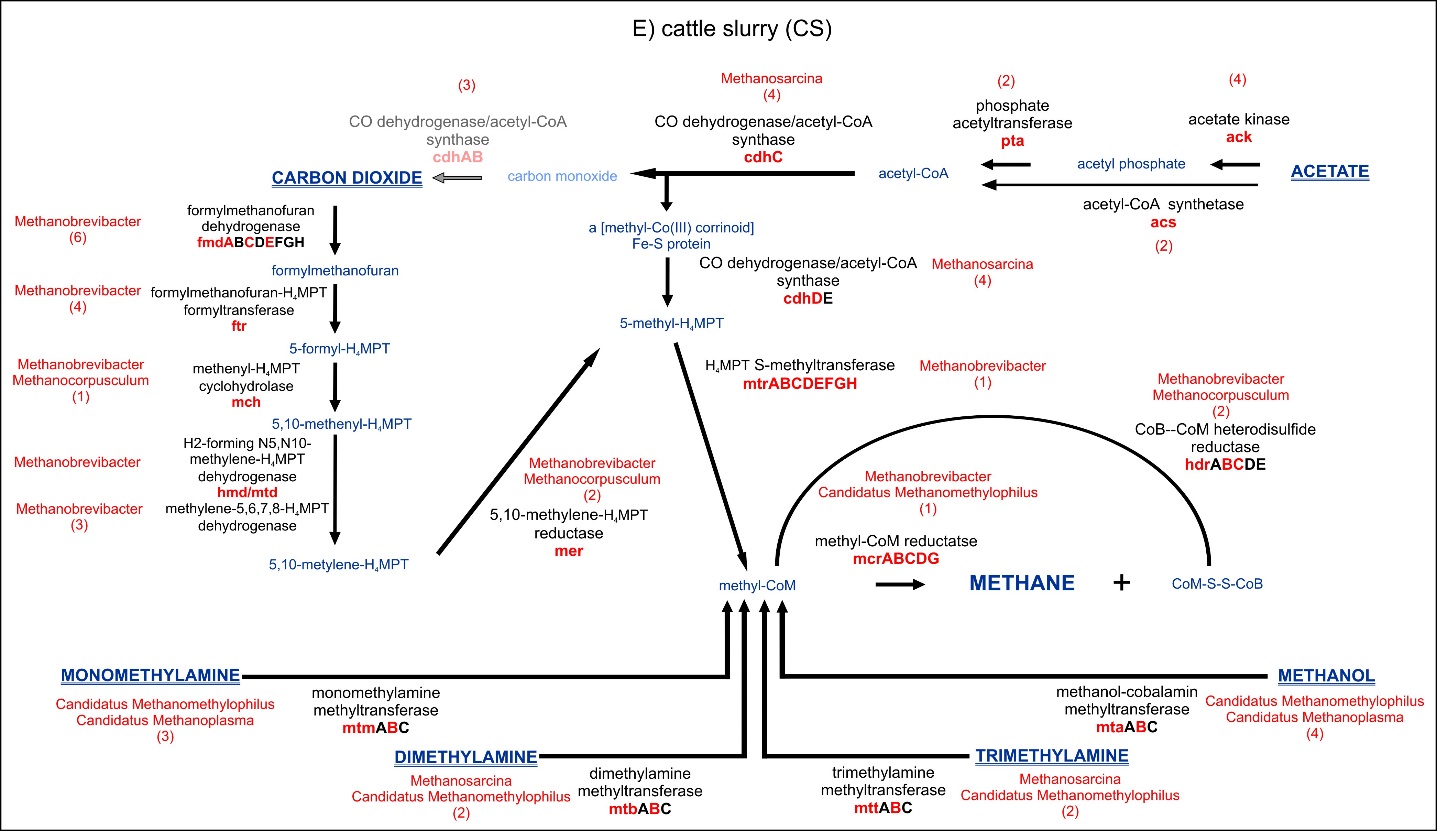

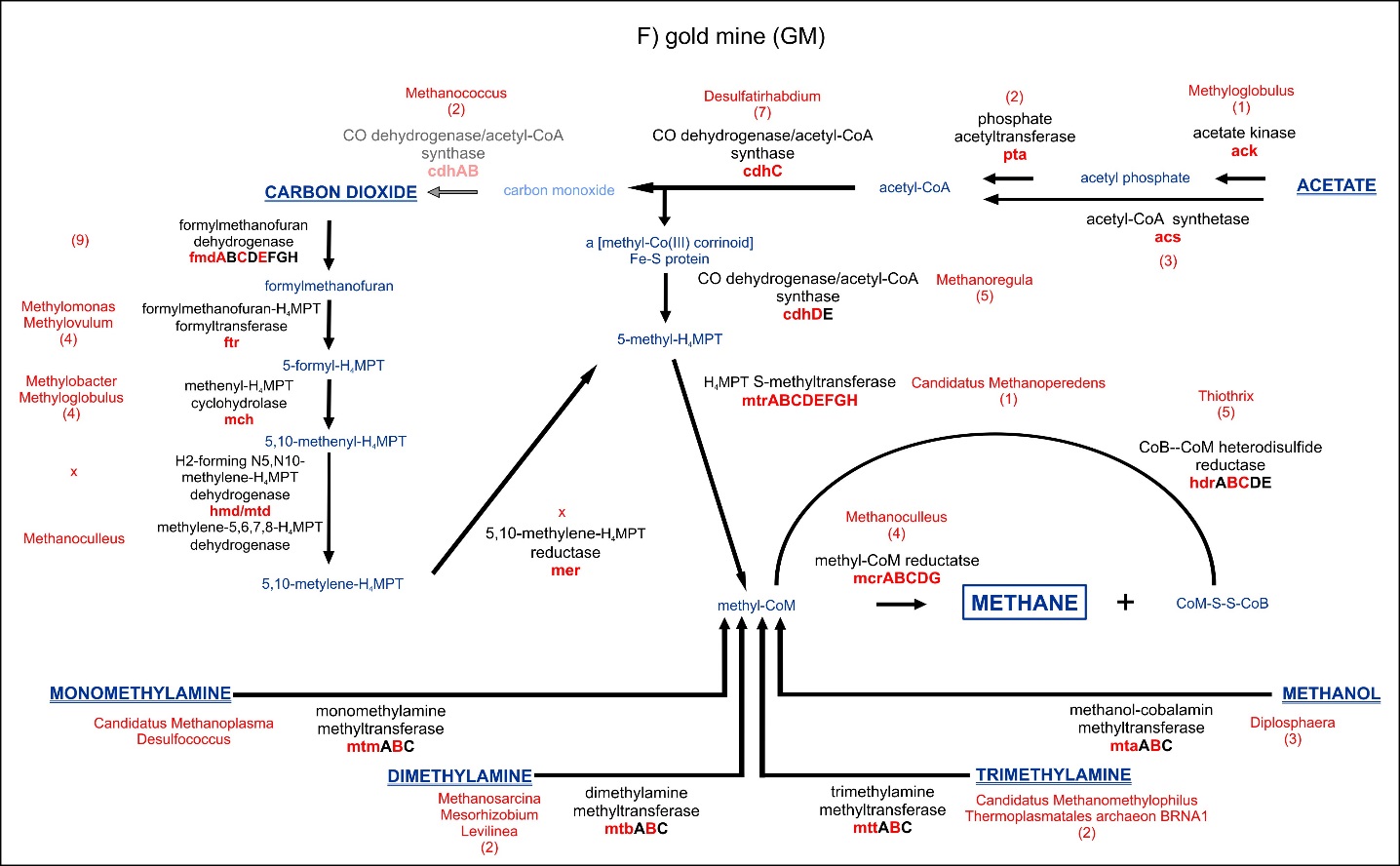

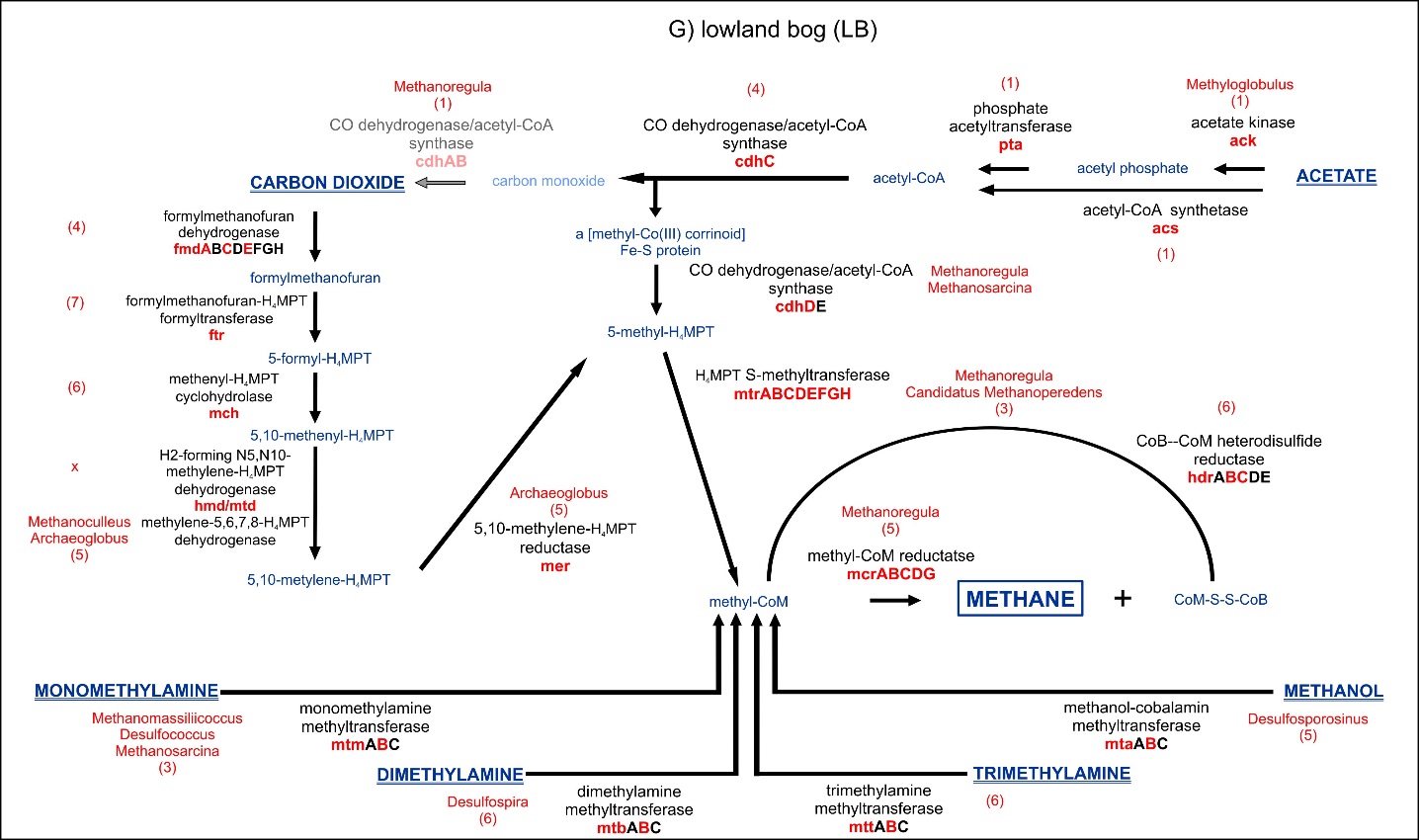
**

**
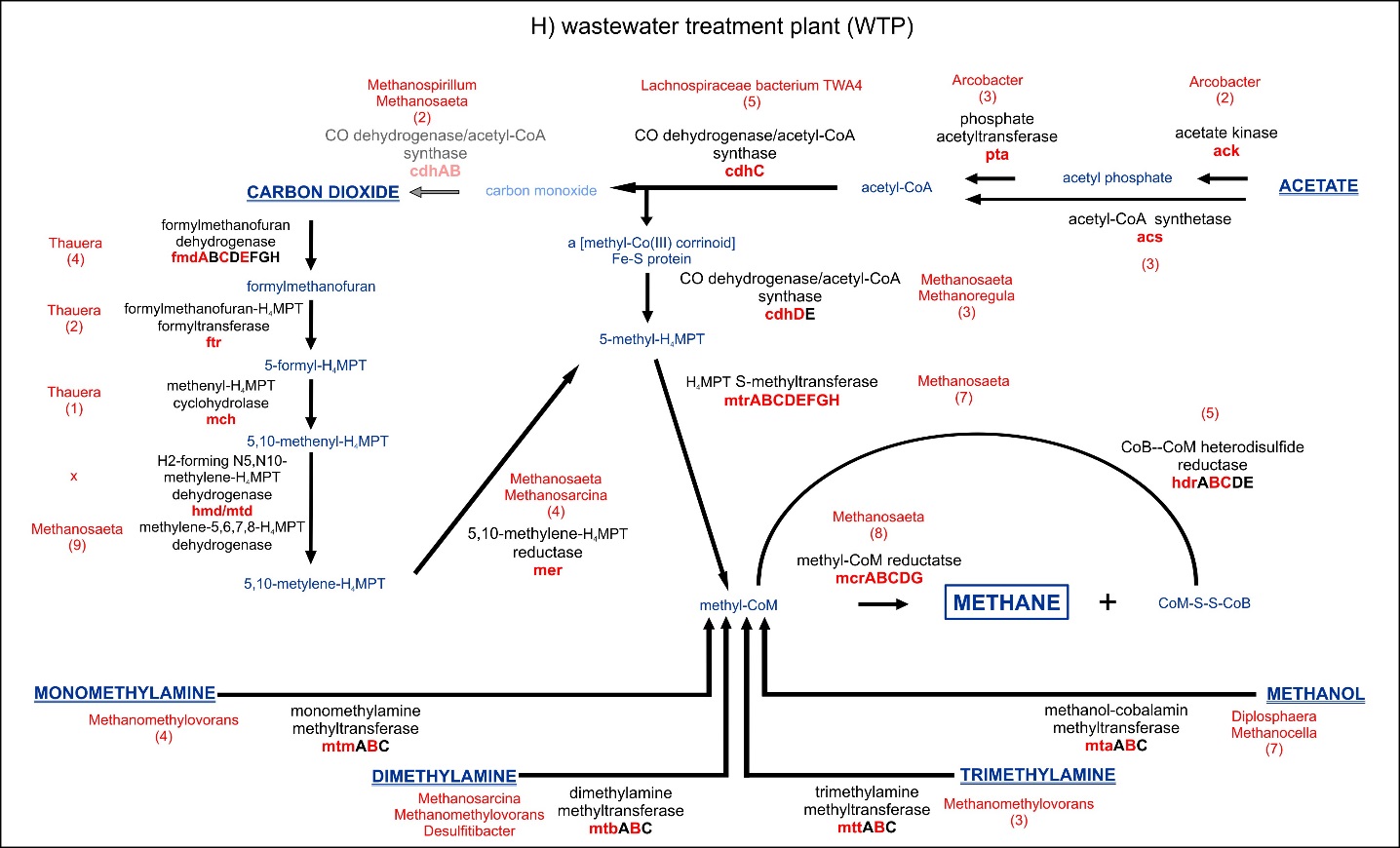
**

**
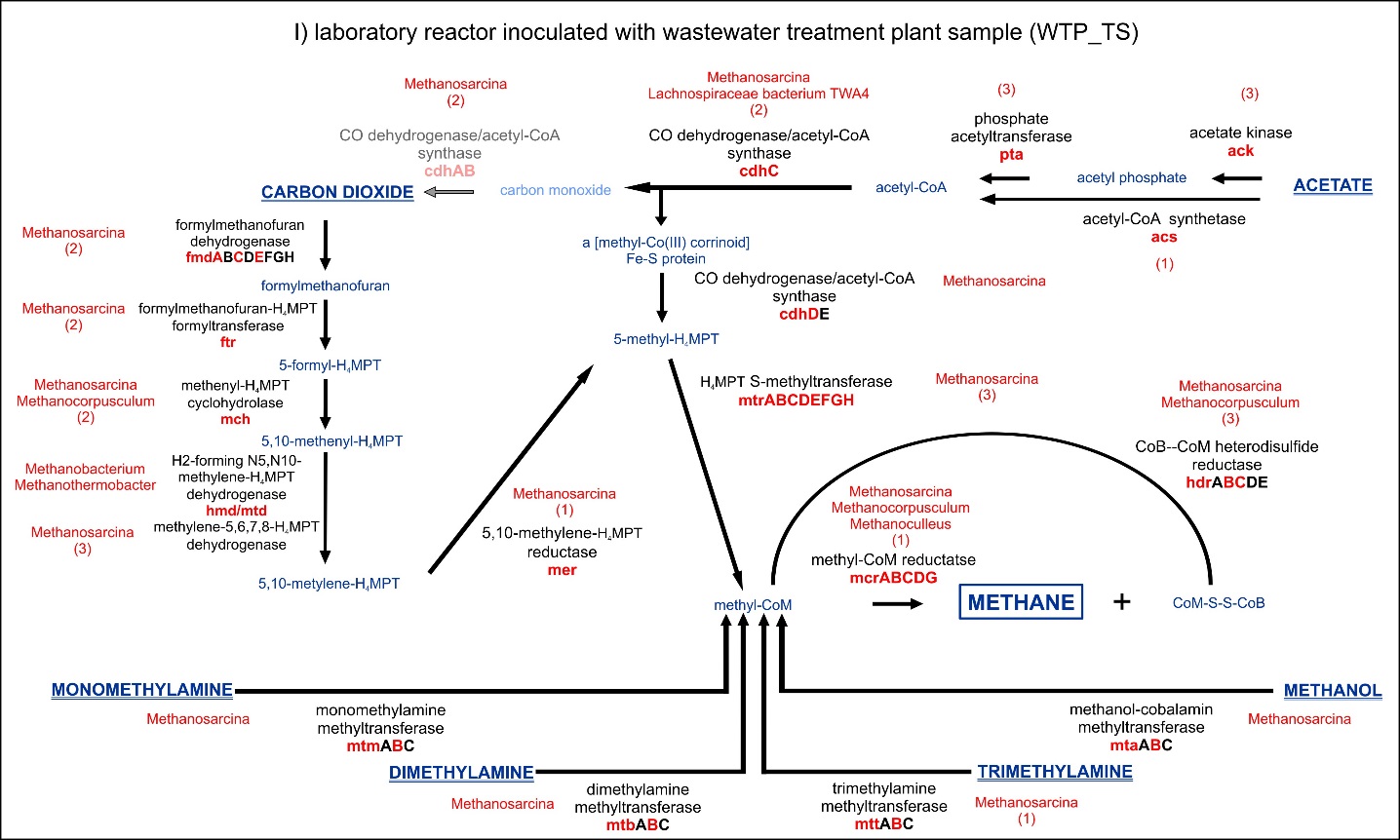
**

**Fig. S3**

Overviews of methanogenesis pathways highlighting the key microorganisms (identified based on MetAnnotate assignments of the genes marked in red) for: A) agricultural biogas fermenter (ABF); B) laboratory reactor inoculated with the agricultural biogas fermenter sample (ABF_TS); C) agricultural biogas hydrolyzer (ABH); D) cattle manure (CM); E) cattle slurry (CS); F) gold mine (GM); G) lowland bog (LB); H) wastewater treatment plant (WTP); I) laboratory reactor inoculated with the wastewater treatment plant sample (WTP_TS). Only genera with hits above 15% were shown with a name. In brackets, the number of microorganisms with hits in the range of 5-15% was indicated. The x sign indicates that sequences for a given enzyme were not detected in metagenomic data by MetAnnotate. The initial substrates for methane production and the final product were marked by bold capital letters. Additionally, initial substrates were underlined and the final product was marked with a frame.


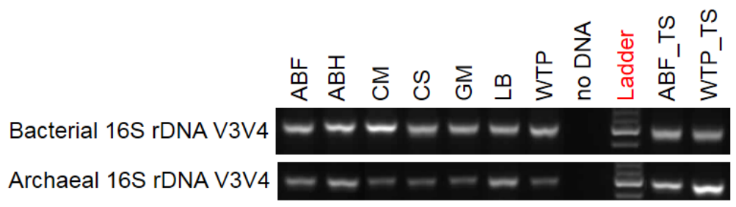


**Fig. S4**

PCR reaction control on isolated metagenomic DNA with primers specific to bacterial and archaeal 16S rDNA variable region V3-V4.
